# Immobilized Copper-Substituted Keggin POM on Graphene Oxide for Highly Selective Heterogeneous Catalysis of Starch Hydrolysis

**DOI:** 10.1021/acsomega.6c00419

**Published:** 2026-06-03

**Authors:** Langson Chilufya, Bright Chilikwazi, Beraat Umur Kaya, Mehtap Emirdag-Eanes

**Affiliations:** † Department of Chemistry, Faculty of Science, 52972 Izmir Institute of Technology, Gülbahçe Campus 35430 Urla, İzmir 35050, Turkey; ‡ Department of Pure and Applied Chemistry, School of Natural Sciences, 247511 University of Zambia, P.O. Box 32379, Lusaka 10101, Zambia

## Abstract

Polyoxometalates (POMs) are bifunctional acid–redox catalysts with significant potential for biomass valorization. Herein, we investigate the heterogeneous catalytic studies of an immobilized 4,4′-bipyridine Cu-substituted Keggin POM, (4,4′-bpyH_2_)_2_(4,4′-bpyH)­[PCuW_11_O_39_]·H_2_O (Bp-PCuW_11_), anchored onto graphene oxide (GO) via ball milling. The Bp-PCuW_11_/GO nanohybrid proved to be an efficient solid acid heterogeneous catalyst for starch hydrolysis, resulting in the formation of glucose as a variable compound. Under optimized hydrothermal conditions, a superior catalytic performance was achieved with 92% starch conversion, 82% glucose yields, and excellent selectivity of 95%, coupled with over five recyclability runs. The Density functional theory (DFT) simulation revealed that the enhanced performance is attributed to the synergistic acid–redox behavior of PCuW_11_ and GO, which enables efficient proton transfer and improved substrate accessibility. This work provides new insights into the rational design of POM–carbon hybrid catalysts for sustainable biomass valorization and other environmentally relevant catalytic transformations.

## Graphical abstract

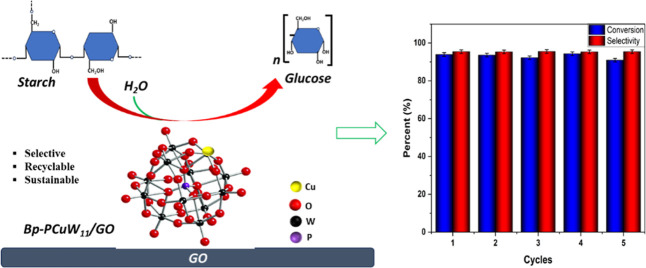

## 1 Introduction

In recent years, the global energy crisis related to the depletion of nonrenewable fossil fuel reserves and the deteriorating quality of the environment has been escalating rapidly and persistently.^1^ Therefore, many countries have adopted renewable and ecologically sustainable energy sources, which is unavoidable.^2,3^ Biomass, a globally abundant and renewable resource, has the potential to emerge as a viable alternative feedstock for the sustainable production of chemicals and fuels.^4^ Starch, a significant component of several biomass sources, is widely found in a range of agricultural byproducts and staple food residues, including, but not limited to, corn, potatoes, rice, and wheat. Starch is a polysaccharide of glucose, predominantly constituted of linear amylose (20–25%) and its branched counterpart, amylopectin (75–80%), which are arranged with 1, 4-and 1, 6-α-glycosidic linkages, respectively.^5^ The depolymerization of starch into monosaccharides, such as glucose, constitutes a significant foundation for producing a wide range of chemicals, fuels, pharmaceuticals, and food products.^6,7^

The catalytic hydrolysis of starch with high selectivity is recognized as a pivotal technological advancement for the valorization of renewable biomass resources. This process involves the protonation of glycosidic oxygen, which lowers the activation energy for bond cleavage, resulting in the production of oligosaccharides and monosaccharides.^8,9^ To date, a significant amount of scientific effort has been dedicated to conventional acid-catalyzed hydrolysis methodologies, which predominantly utilize potent mineral acids, such as H_2_SO_4_ and HCl.^10^ Despite their effectiveness, these mineral acids present numerous challenges, including equipment corrosion, complex catalyst recovery, and the production of an acidic waste stream. Another alternative in catalytic hydrolysis is the use of biological enzymes, which can achieve elevated hydrolytic outputs under moderate conditions. However, they have notable drawbacks, including limited enzymatic activity, elevated costs associated with enzyme procurement, and challenges related to separation due to their solubility in aqueous environments.^11–14^ These limitations have sparked significant research interest in developing heterogeneous solid acid catalysts that are efficient, selective, and environmentally benign for starch hydrolysis and related biomass transformations.^15,16^

Among these numerous heterogeneous solid acid catalysts, polyoxometalates (POMs), a distinctive class of discrete metal–oxygen anionic clusters, have emerged as highly efficient catalysts owing to their strong Brønsted and Lewis acidity, redox versatility, and structural tunability at the molecular level.^17^ Among a wide range of applications of POMs, catalysis stands out as the most appealing due to its remarkable adaptability in altering redox characteristics, ease of manipulation, environmental sustainability, nonhazardous nature, and experimental straightforwardness. Indeed, compared to cellulose, a very few catalysts for the depolymerization of starch to glucose have been reported in the past few years. Hydrothermal starch-conversion conditions utilizing POM solid acid catalysts constitute an environmentally sustainable approach that has garnered significant attention in contemporary research efforts. For instance, Cheng et al.^18^ designed a micellar heteropoly acid catalyst, (C_16_H_2_PW), that exhibited outstanding catalytic performance for the hydrolytic degradation of polysaccharides. In the context of starch hydrolysis, the catalytic ability of C_16_H_2_PW resulted in glucose yields and selectivity of 82% and 86%, respectively. We recently synthesized and characterized two POMs, a Keggin, (4,4′-bpyH_2_)_3_[PW_12_O_40_]­2·3H_2_O, and a Wells-Dawson, (4,4′-bpyH_2_)_3_(4,4′-bpyH)_1.75_[Cu­(bpy)_2_]_0.25_[H_2_P_2_W_18_O_62_]_2_. When these two were utilized as solid catalysts for starch hydrolysis under hydrothermal conditions of 150 °C for 5 h, they both achieved a glucose yield of more than 90%.^19^ Notably, in these two works, the organic counterion assemblies of POMs enhanced the local acid concentration and substrate adsorption, yielding higher rates than those of simple pristine POMs, which illustrates that local acidity and adsorption control the rate and selectivity. The ability of POMs to act as both proton donors and electron acceptors has enabled them to catalyze a wide range of acid-catalyzed and redox reactions.^20^ However, pristine POMs often exhibit high solubility in aqueous and polar media. Additionally, they tend to aggregate due to their limited surface area. This frequently results in leaching, limited recovery, and long-term catalytic performance in aqueous systems.^21,22^

Recognizing these drawbacks, POMs are commonly immobilized onto solid supports, such as metal–organic frameworks, metal oxides, zeolites, silica, or carbon-based materials, to enhance their surface accessibility and stability.^11,23–27^ Graphene, characterized as a two-dimensional (2D) monolayer of hexagonally arranged carbon atoms, is emerging as a prominent subject of investigation among the various constituents of the carbon materials family.^28,29^ Graphene oxide (GO), with its large specific surface area, abundant oxygen-containing groups (−COOH, −OH, and −O−), and high chemical stability, provides an ideal substrate for POM anchoring.^30^ The strong electrostatic and covalent interactions between POM anions and GO functional groups ensure uniform dispersion of active sites, which may minimize leaching during catalysis. Furthermore, the conductive and layered structure of GO facilitates electron transfer, enhancing the catalytic kinetics of POM-based hybrids.^31^

Many POMs are inherently water-soluble and often undergo desorption under hydrothermal starch conversion conditions unless firmly immobilized.^26,32^ However, long-term leaching data for POM catalysts anchored on GO during starch hydrolysis remain scarce. Since native starch granules and semicrystalline polysaccharides require catalysts with accessible acid sites, rational tuning of the hydrophilic–hydrophobic balance around POMs on the GO surface is crucial for achieving efficient and stable catalytic performance. The synergistic effect between POM and GO can significantly enhance catalytic performance through improved acid strength distribution, reduced POM leaching, increased surface area, and better mass transport.^33^ In the context of starch hydrolysis, these POM/GO nanohybrid catalysts may facilitate the selective cleavage of glycosidic bonds, yielding higher glucose yields with minimal formation of byproducts. To the best of our knowledge, Cu-substituted POM immobilized on GO has not been previously reported for starch hydrolysis.

Herein, we report the synthesis and characterization of a nanohybrid of a 4,4′-bipyridine Cu-substituted Keggin POM, (4,4′-bpyH_2_)_2_(4,4′-bpyH)­[PCuW_11_O_39_]·H_2_O (Bp-PCuW_11_) grafted onto GO via a facile ball milling procedure. In comparison with other heterogeneous catalysts for the selective hydrolysis of starch, the Bp-CuPW_11_/GO nanohybrid demonstrated superior catalytic efficiency, high selectivity toward glucose formation, and excellent reusability. Under the optimized conditions, the Bp-PCuW_11_/GO catalyst achieved a 92% starch conversion, 82% glucose yield, and excellent selectivity of 95%, coupled with recyclability over five consecutive runs. The superior catalytic efficiency is attributed to the synergistic effect between the Brønsted acidic POM units and the hydrophilic, high-surface-area GO support, which collectively enhances proton transfer, substrate adsorption, and mass transport during the hydrolysis process. The hybrid catalyst maintained structural integrity after repeated use, confirming its robustness and low leaching tendency. The density functional theory (DFT) calculations demonstrated that the improved catalytic performance is attributed to the uniform dispersion of Bp-CuPW_11_ POM clusters on the GO surface, which facilitates better exposure of active sites and effective interaction with starch molecules. This work demonstrates that rational integration of POMs with graphene-based support can yield high-performance solid acid catalysts with tunable acidity, stability, and selectivity. Additionally, this study demonstrates that rational integration of POMs with GO can yield robust, high-performance solid acid catalysts for sustainable biomass conversion processes.

## 2 Results and Discussions

### 2.1 Materials Characterization

The Bp-PCuW_11_/GO nanohybrid was synthesized through a two-step process, as illustrated in Scheme 1, involving a hydrothermal and ball milling procedure. In a previous study from our lab, we demonstrated the synthesis and characterization of an organic–inorganic hybrid (Bp-PCuW_11_) with a one-dimensional chain structure of monolacunary copper-substituted Keggin anion [PCuW_11_O_39_]^5–^, under optimized hydrothermal conditions.^34^ Meanwhile, graphene was oxidized to GO via the Hummers modified method and incorporated into the hybrid by ball milling, yielding the final nanohybrid, Bp-PCuW_11_/GO.^35,36^ The PXRD pattern in Figure 1a shows characteristic Keggin peaks of Bp-PCuW_11_ at 9.69°, 11.8°, 22.6°, and 29.8°. The experimental PXRD pattern of Bp-PCuW_11_ was compared with the simulated pattern generated from the reported crystallographic data using mercury program (Figure S1).^34^ The close agreement in diffraction peak positions confirms the successful formation of the targeted Keggin-type structure. Minor variations in relative intensities are attributed to preferred orientation effects and reduced crystallite size. The Bp-PCuW_11_ peaks which remain visible in the Bp-PCuW_11_/GO nanohybrid, confirming that the Keggin structure is preserved without significant degradation.^37^ Additionally, the broad peak of graphitic sp^2^ that characterizes the GO at 26.1° (002) is obtained in the nanohybrid, indicating that the Bp-PCuW_11_ was successfully embedded in the GO surface.^38^

**Scheme 1:**
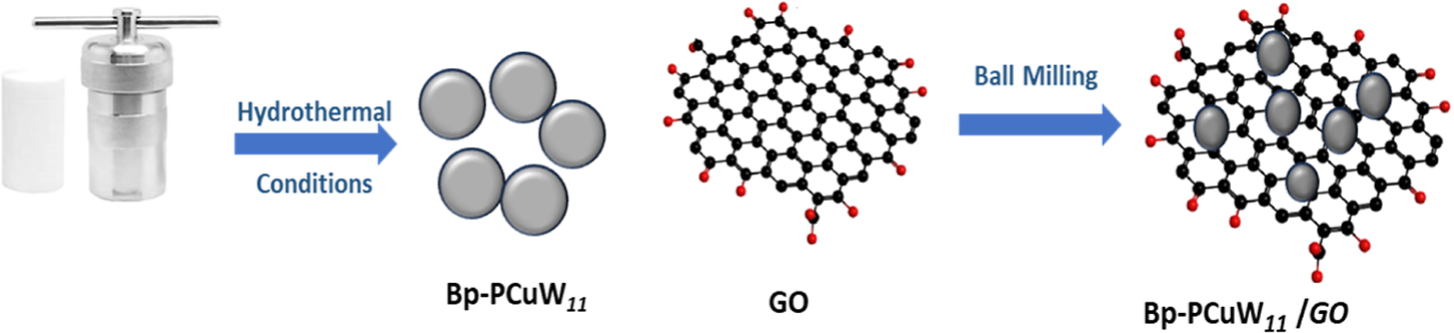
Illustration of the Two-step Synthesis of Bp-PCuW_11_/GO Nanohybrid^a^

a Adapted with permission from ref 50 Copyright 2022, Springer Nature.

**Figure 1:**
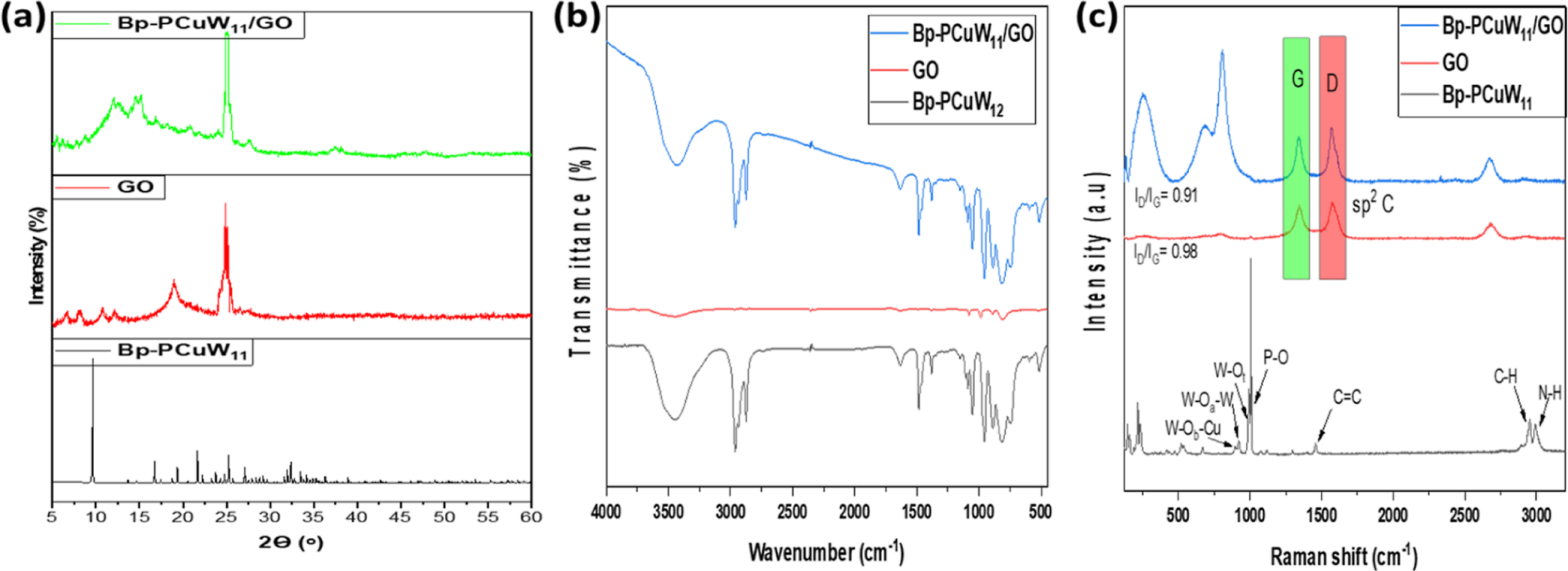
Spectra patterns of Bp-PCuW_11_, GO, and Bp-PCuW_11_/GO nanohybrid in (a) powder XRD, (b) FT-IR, and (c) Raman.

Although the GO (002) reflection partially overlaps with one of the Bp-PCuW_11_ diffraction regions near 26°, the distinction between the two phases is evident from the difference in peak profiles, where GO contributes a broad diffuse band while Bp-PCuW_11_ retains sharp crystalline reflections. Furthermore, several additional characteristic peaks of Bp-PCuW_11_ remain clearly observable in the hybrid, confirming preservation of the inorganic framework after immobilization. Notably, the intensity of the GO (002) reflection decreases and becomes slightly broadened in the hybrid material, suggesting partial disruption of GO interlayer stacking due to the incorporation of Bp-PCuW_11_ clusters. Worth noting, are the PXRD patterns (Figure S2) that reveal clear structural differences between the manually mixed Bp-PCuW_11_ + GO and the ball-milled Bp-PCuW_11_/GO nanohybrid. The manually mixed sample exhibits numerous sharp and intense diffraction peaks in the 10–35° (2θ) region, confirming the preserved crystallinity of Bp-PCuW_11_ after physical blending. In contrast, the ball-milled composite shows markedly reduced peak intensities with significant peak broadening and partial suppression of characteristic reflections. Such suppression or broadening of diffraction peaks is commonly reported for polyoxometalate/carbon hybrids, where strong interfacial interactions and nanoscale dispersion within conductive carbon matrices disrupt long-range order while preserving the local POM structure.^39–43^ This attenuation of long-range order indicates decreased crystallinity and structural distortion induced by mechanochemically treatment, suggesting enhanced dispersion and stronger interfacial interaction between Bp-PCuW_11_ and GO.

Figure 1b compares the FT-IR spectra of Bp-PCuW_11_/GO nanohybrid and its individual components. The presence of distinct Keggin POM IR bands for the formation of Bp-PCuW_11_ at 820, 912, 992, and 1087 cm^–1^, corresponding to the *v*(P–O_a_), terminal *v*(W–O_t_), corner sharing *v*(W–O_b_-W), and edge sharing *v*(W–O_c_-Cu), respectively. This indicated that the nanohybrid retains the structural integrity of the Cu-substituted Keggin PCuW_11_ after formation.^44^ The Pyridine vibrations at 1628 cm^–1^ emanating from the *v*(C=C) in 4,4′-bipyridine can also be observed in the nanohybrid, though it was suppressed by the vibration peaks coming from the *v*(C–C) and *v*(C–O) from the GO.^45,46^ In contrast, the manually mixed Bp-PCuW_11_ + GO spectrum displays sharper and more distinct individual bands corresponding to both components (Figure S3). The characteristic Keggin vibrations are clearly resolved, indicating minimal structural interaction. The GO-related bands, particularly C=O stretching (1720 cm^–1^) and C–O vibrations (1050–1250 cm^–1^), appear more independent and less perturbed. This suggests that ball milling processing promotes enhanced interaction between Bp-PCuW_11_ and GO rather than simple physical blending.

Meanwhile, the Raman spectra in Figure 1c corroborate well with the FT-IR spectra with characteristic peaks vibration observed for Bp-PCuW_11_ at 1023, 1006, 987, 840, and 778 cm^–1^, corresponding to *v*(P–O_a_), *v*(W–O_t_), *v*(W–O_b_-W), and *v*(W–O_c_-W) stretching vibrations, respectively.^47^ These IR bands, together with the graphitic sp^2^ hybridized carbon in GO at 1342 cm^–1^ (D-band), and sp^2^-hybridized graphitic carbon at 1579 cm^–1^ (G-band), are discernible in the Bp-PCuW_11_ /GO nanohybrid.^48,49^ The calculated *I*_D_/*I*_G_ ratio for GO is 0.98, indicating a relatively high defect density typical of oxidized graphene materials. After hybrid formation, the Bp-PCuW_11_/GO composite retains the D and G bands; however, the *I*_D_/*I*_G_ ratio decreases slightly to 0.91. This reduction suggests modification of the defect environment upon incorporation of Bp-PCuW_11_. The change in *I*_D_/*I*_G_ ratio can be attributed to interfacial interaction between the oxygenated functional groups of GO and the POM clusters, which may partially alter defect distribution and electronic structure within the graphene framework. These observations from PXRD, FR-IR and Raman collectively confirm successful hybrid formation through interfacial interaction between Bp-PCuW_11_ and GO rather than simple physical mixing.

The SEM and STEM were used to analyze the morphology of the Bp-PCuW_11_/GO nanohybrid. The SEM image in Figure 2a shows that the GO layers have been successfully synthesized with a spherical morphology, indicating that the Bp-PCuW_11_ structure is assembled on their surface. Unlike the SEM image in Figure S4­(a) for the Bp-PCuW_11_ structure, which showed a relatively smooth surface, the nanohybrid exhibited a rough surface morphology. This observation was also consistent with the STEM image at 10 μm, as shown in Figure 2b, which displays uniformly distributed, sparkly dots corresponding to the Bp-PCuW_11_ structure. The STEM image in Figure S4­(b) at a higher magnification of 300 nm further revealed the rough surface of the nanohybrid, which is attributed to the presence of GO, absent in the pristine material. The morphologies of this nanohybrid correlated well with those reported for carbonaceous POM-based nanohybrids, which show rough surfaces after nanohybridization.^51,52^

**Figure 2:**
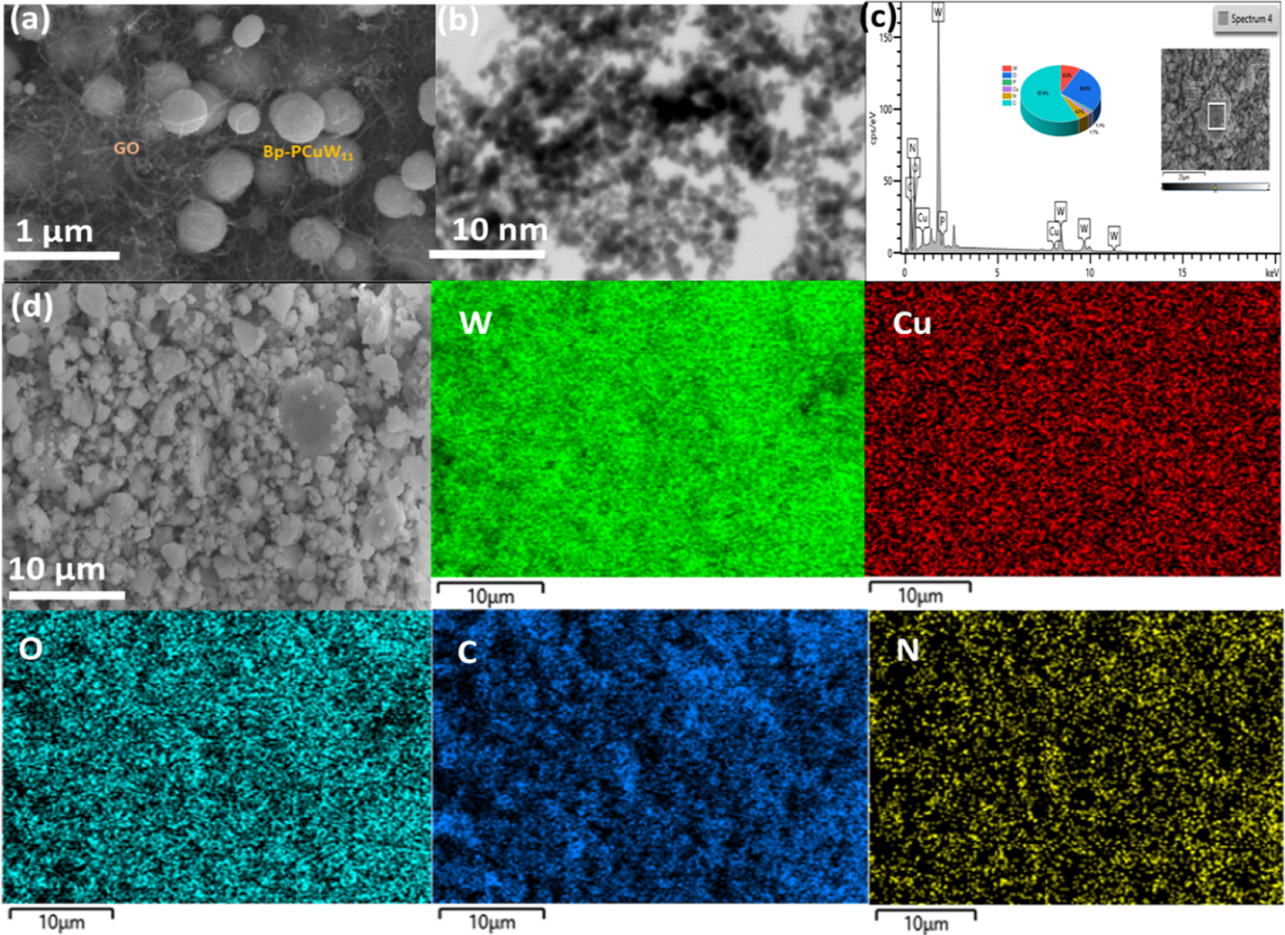
Morphology and elemental analysis of Bp-PCuW_11_/GO nanohybrid; (a) SEM images 10 μm, (b) STEM images at 10 μm. (c) EDX spectra showing elemental proportions, (d) EDS elemental mappings with even distribution of elements.

EDX analysis in Figure 2c revealed the elemental composition and their relative proportions, with strong signals for W (8.19%) and O (26.91%), as well as P (1.08%) and Cu (1.69%), consistent with the [PCuW_11_O_39_] anionic framework. The detection of N (4.68%) signals confirms the presence of the protonated 4,4′-bypridine counter-cations. Additionally, the prominent peaks of C (57.44%) originating from the GO and counter-cations confirm the successful anchoring of the Bp-PCuW_11_ structure onto the GO. Moreover, the EDX elemental mapping in Figure 2d revealed distinct signals for P, W, O, N, Cu, and C with uniform dispersion, verifying that the Bp-PCuW_11_ structure was successfully incorporated on the GO framework.

The thermal stability in TGA analysis, as shown in Figure 3a, indicates that the Bp-PCuW_11_ structure exhibits a gradual mass loss within the temperature spectrum of roughly 103–267 °C, which is predominantly ascribed to the elimination of adsorbed moisture. This was followed by the sequential removal of the three Bp groups, resulting in an average weight loss of 19% up to 266 °C.^53^ In contrast, the Bp-PCuW_11_/GO nanohybrid exhibited a sharp weight loss above 540 °C, attributed to the decomposition of the GO network (44%), which was converted into CO or CO_2_ residues.^54^ The DTA curve correlates with the phase transition temperatures of water, pyridinyl carbon framework, and GO at 121, 238, and 572 °C, respectively.

**Figure 3:**
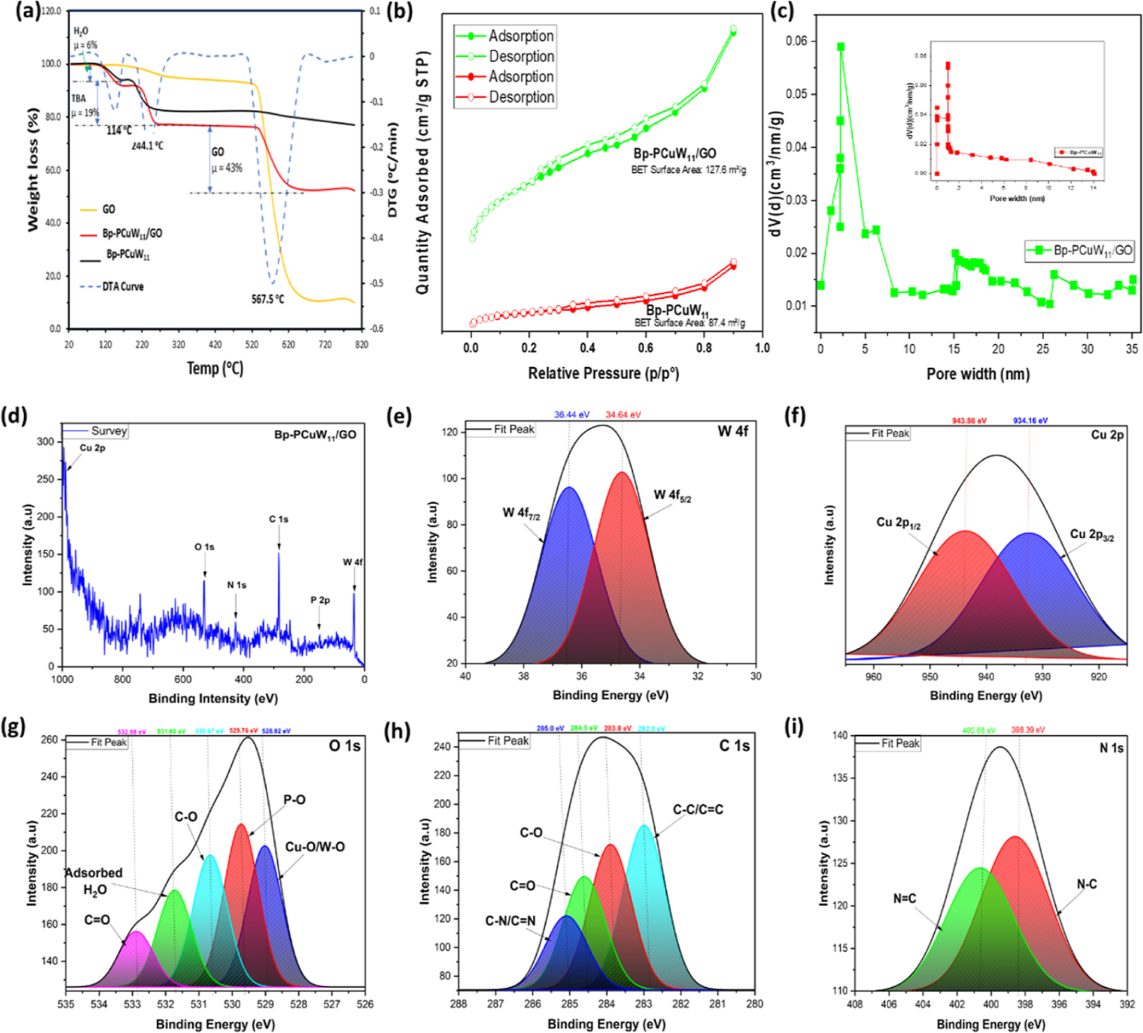
(a) The TGA curves of Bp-PCuW_11_, GO, Bp-PCuW_11_ /GO nanohybrid, and the DTG of the as-prepared Bp-PCuW_11_ /GO nanohybrid. (b) N_2_ adsorption/desorption isotherm of Bp-PCuW_11_ and Bp-PCuW_11_/GO nanohybrid. (c) Pore size distribution of Bp-PCuW_11_ and Bp-PCuW_11_/rGO nanohybrid. (d) The XPS survey spectrum of Bp-PCuW_11_/GO. The high resolution deconvoluted spectra of (e) W 4f, (f) Cu 2p, (g) O 1s (h) C 1s and (i) N 1s.

The Brunauer–Emmett–Teller (BET) nitrogen adsorption isotherms were used to analyze the surface area and pore size of the Bp-PCuW_11_/GO nanohybrid. As illustrated in Figure 3b, the N_2_ adsorption–desorption isotherm classification was of type IV, characterized by hysteresis loops, indicating a mesoporous architecture. The higher BET surface area of the Bp-PCuW_11_ /GO nanohybrid (127.6 m^2^ g^–1^) compared to that of the pure Bp-PCuW_11_ structure (87.4 m^2^ g^–1^) is due to the presence of the GO structure. This suggests that incorporating GO can increase its specific surface area and enhance the exposure of active sites, leading to improved catalytic performance by the Bp-PCuW_11_/GO nanohybrid.^55^ Furthermore, the mesoporosity was confirmed by the pore size distribution shown in Figure 3c. Strong and sharp peaks were observed just above the pore width of 2.7 nm, which indicates a significant presence of mesopores from the GO surface. The presence of large pores facilitates starch substrate interactions with the active sites of the Bp-PCuW_11_/GO catalyst, thereby accelerating the reaction kinetics and enhancing the overall efficacy of the catalytic mechanism.^56^ This was further consolidated by the increased micropore volume features of Bp-PCuW_11_/GO (0.25 cm^3^ g^–1^), which facilitate catalyst interaction with the starch substrate, thereby maximizing catalytic efficiency and yield. Table S1 summarizes and compares the BET surface area, pore size distribution, and micropore volume of the nanohybrid and the pristine POM.

To evaluate the surface chemical states and valence bonding of the Bp-PCuW_11_/GO nanohybrid, X-ray photoelectron spectroscopy (XPS) analysis was conducted. The XPS survey spectrum in Figure 3d reveals the presence of the elements and their orbitals, specifically W 4f, P 2p, C 1s, N 1s, O 1s, and Cu 2p, with corresponding peaks at approximately 35, 137, 286, 400, 535, and 963 eV, respectively. The successful anchoring of Bp-PCuW_11_ onto GO is confirmed by the higher intensity of the C 1s peaks in the nanohybrids compared to the survey spectra of pristine Bp-PCuW_11_. The spectrum in Figure 3e presents distinct W 4f doublet peaks with characteristic binding energies of 36.44 eV (W 4f_7/2_) and 34.64 eV (W 4f_5/2_), attributed to the dominant W^6+^ oxidation state in Bp-PCuW_11_. Meanwhile, the Cu 2p spectrum in Figure 3f exhibits a doublet peak corresponding to Cu^2+^ 2p_1/2_ (943.86 eV) and Cu^+^ 2p_3/2_ (934.16 eV).

By basing all binding energies on the reference, the deconvoluted spectrum of the C 1s in Figure 3g gives five distinct chemical environments at 285.01, 284.47, 283.76, and 282.81 corresponding to C–N/C=N, C=O, C–O, and C–C/C=C bonds, respectively.^57^ The overlapping O 1s signals from both Bp-PCuW_11_ and GO are confirmed in Figure 3h with fitted peaks at 532.88, 531.68, 530.67, 529.76, and 528.92 eV, corresponding to C=0, O–H (adsorbed water), C–O, P–O, and Cu–O/W–O, respectively.^58,59^ The N 1s spectrum in Figure 3i exhibits two characteristic bonding configurations, attributed to the pyridinic N=C (400.39 eV) and pyrrolic N–C (398.39 eV), indicating the 4,4′-bipyridine was intact in the nanohybrid. Compared to the pristine Bp-PCuW_11_ (Figure S5 and Table S2), there is a slight shift in the binding energies, except for the C 1s in the nanohybrid after anchoring on GO. Generally, the overall shift of distinctive peaks to higher binding energies in the nanohybrid unequivocally evidence the successful anchoring of Bp-PCuW_11_ on GO. This typically occurs in nanohybrids of POM with carbon allotropes due to electron transfer between the two, resulting in altered electronic environments.^58,60^ The XPS analysis data correlate well with the EDX, FT-IR, and Raman results, confirming the presence of elements and their chemical bonding.

### 2.2 Evaluation of Catalytic Activity

The nature and potency of the catalyst’s acidic properties exert a significant influence on the hydrolysis of starch. The pathway of this hydrolysis reaction follows the Brønsted acidic sites in traditional POM acid catalysts that effectively protonate the oxygen glycosidic bond (C–O–C), with subsequent nucleophilic attack on the adjacent carbon. A cascade of reactions involving protonation and nucleophilic attack facilitates the ring opening, resulting in the formation of a linear monosaccharide, such as glucose, as demonstrated in Scheme 2. This polysaccharide hydrolysis is a crucial process for producing glucose, a major platform for the synthesis of numerous value-added products and chemicals, including fructose, glyceraldehyde, glycolic acid, and formic acid, which are mainly required in the food and pharmaceutical industries.

**Scheme 2:**
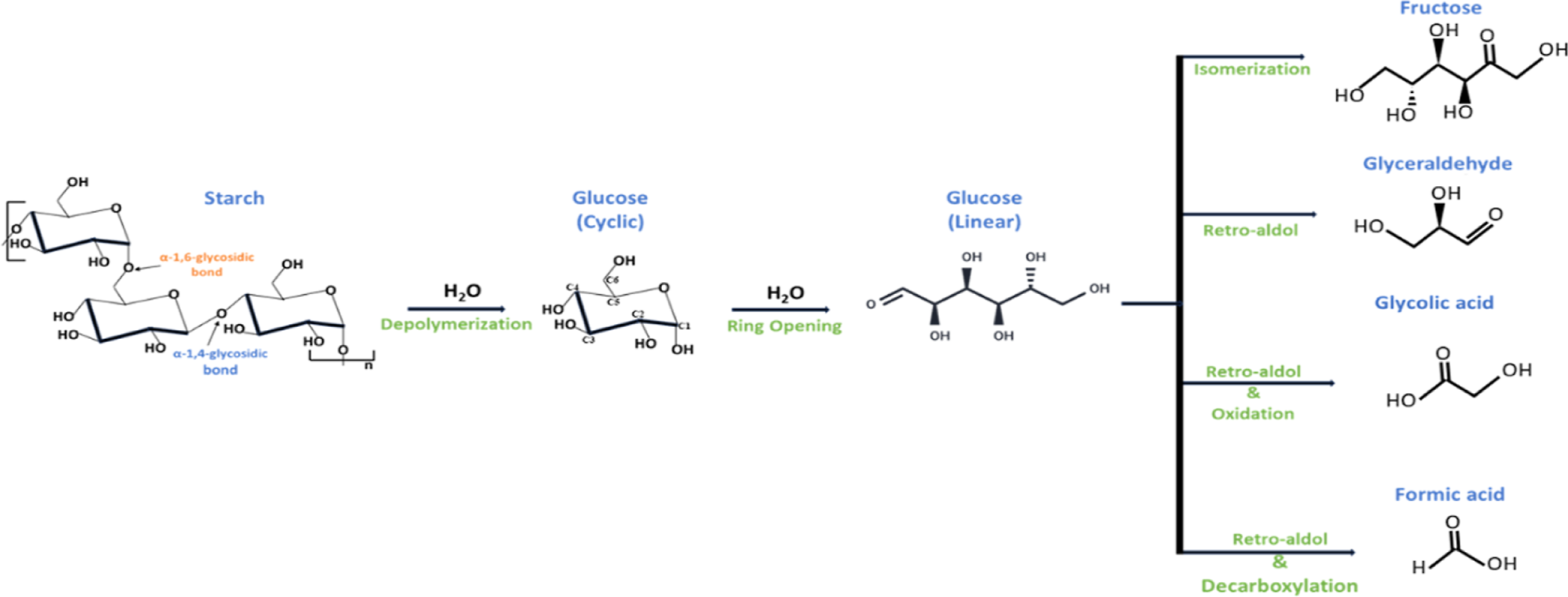
Catalytic Hydrolysis of Starch to Glucose and Subsequent Reactions to Give Value-Added Products and Chemicals

This study investigates the catalytic performance of Bp-PCuW_11_ and Bp-PCuW_11_/GO catalysts in the hydrolysis of starch under specific reaction conditions, including 0.1 g of starch and 10 mg of catalyst at 150 °C in 7 mL of distilled water. Initially, the effect of different mass ratios of GO to Bp-PCuW_11_ was studied. The mass ratio of 1:20, corresponding to the ball mill mixing of 0.25 g of GO and 5.0 g of Bp-PCuW_11_, yielded a maximum of 90% as shown in Figure S6. This mass ratio was adopted for all analyses of starch hydrolysis. The hydrolysis reaction, conducted without a catalyst, served as a control experiment to assess the catalytic properties. The Time-dependent UV–vis absorption spectra for starch hydrolysis using the Bp-PCuW_11_/GO catalyst are shown in Figure 4a. The hydrolysate filtrate was analyzed for reducing sugar concentration, using the DNS (3,5-dinitrosalicylic acid) method, which revealed varying intensities corresponding to glucose concentrations (Figure S7).^61,62^ The catalyst demonstrates hydrolysis efficiency, with a 5 h time giving the highest absorbance at a wavelength of λ = 554.2. The 5 h time was adopted for complete hydrolysis. The unreacted starch was removed by centrifugation, and the formation of water-soluble saccharides, specifically glucose, was confirmed by Lugol’s iodine test for starch, as shown in Figure 4b.^19^ This investigation revealed that the glucose generated in this analysis had a linear organic chain, as it contains a terminal aldehyde group, which is reactive to the DNS and Lugol’s solution.

**Figure 4:**
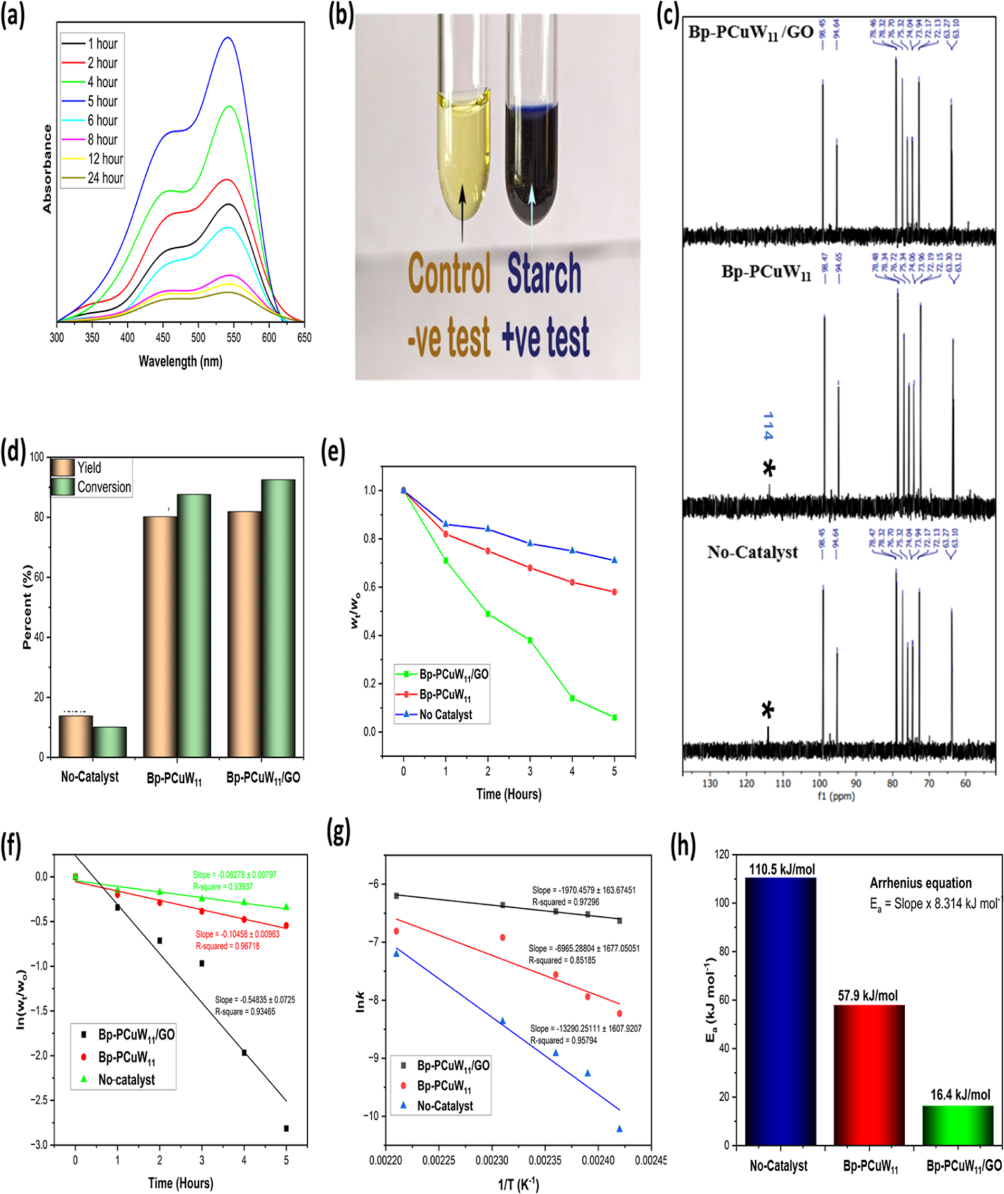
(a) UV–vis Absorption spectra of glucose concentration in the presence of DNS. (b) Lugol’s iodine test for saccharides. Adapted from a photograph courtesy of Thomas Tungnung. Copyright 2021 (c) the ^13^C NMR spectra of glucose after starch hydrolysis. (d) The reaction performance of the reaction with no catalyst and one with Bp-PCuW_11_ and Bp-PCuW_11_/GO catalysts. (e) The catalytic activities for the reaction with no catalyst and one with Bp-PCuW_11_ and Bp-PCuW_11_/GO catalysts. (f) Pseudo-first-order kinetic fitting for starch hydrolysis. (g,h) Arrhenius plots and the corresponding calculated activation energies.

Furthermore, the depolymerization of starch after hydrolysis was also confirmed by ^13^C NMR spectroscopy, as illustrated in Figure 4c, which revealed six different types of carbons, typical of glucose, in the range of 65 to 100 ppm. However, after hydrolysis with the No-catalyst and Bp-PCuW_11_, a notable additional peak at 114 ppm was observed, attributed to the unreacted starch.^26,63^ Interestingly, in the hydrolysis with the Bp-PCuW_11_/GO catalyst, complete depolymerization of starch was observed, with no other side products, such as fructose, glyceraldehyde, glycolic acid, and formic acid, being obtained within the detection limits of the ^13^C NMR analysis. This implied that, the ^13^C NMR spectrum shows signals corresponding exclusively to glucose within the detection limits of the technique, with no additional observable carbon resonances attributable to secondary products. This clearly showed the outstanding selectivity of the Bp-PCuW_11_/GO catalyst for starch hydrolysis.

The selectivity was further assessed, as shown in Figure 4d, where, without any catalyst, less starch was hydrolyzed into water-soluble monosaccharides, specifically glucose, under these reaction conditions. In contrast, catalytic reactions using catalysts Bp-PCuW_11_ and Bp-PCuW_11_/GO exhibited enhanced hydrolysis, displaying superior catalytic performance. The Bp-PCuW_11_/GO catalyst, and without it, the glucose yields reached 82% and 14%, respectively. Compared to Bp-PCuW_11_, catalytic activity is significantly improved when using Bp-PCuW_11_/GO under the same reaction conditions, with a conversion efficiency of 93%. Furthermore, the catalytic activity was analyzed, as shown in Figure 4e, where the Bp-PCuW_11_/GO catalyst exhibited a gradual and steady decrease over time. These results confirm that the observed decrease in reaction rate during a single run is not due to rapid catalyst deactivation but is primarily governed by substrate concentration effects. This is consistent with concentration-dependent hydrolysis kinetics for first order reactions. Although displaying relatively weaker performance compared with the Bp-PCuW_11_ and Bp-PCuW_11_/GO catalysts, the no-catalyst hydrolysis still demonstrated decent formation of glucose, which is somewhat unexpected. This was attributed to the ionic product of H^+^ and OH^–^ ions from water, which can infiltrate the starch macromolecule throughout the hydrothermal reaction mechanism, cleaving the α-1,4 and α-1,6 glycosidic linkages inherent to starch.^64^ Additionally, this demonstrates that hydrothermal conditions are a suitable method for starch hydrolysis and an environmentally benign approach.

To confirm the heterogeneous nature of the catalyst, hot filtration experiments were conducted. As illustrated in Figure S8, the catalyst removal at 2 h resulted in a glucose yield of 32% while the uninterrupted reaction reached 99%. The absence of the Bp-PCuW_11_/GO in the filtrate indicated the heterogeneous character of the catalyst in starch hydrolysis. The catalytic hydrolysis of a polysaccharide was also examined using starch as the model substrate, along with four other nanohybrids synthesized in our lab as heterogeneous catalysts.^39,65,66^ These compounds were K_3_PW_12_W_40_/SWCNT (KPW_12_/SWOH), K_2_PW_12_W_40_/GO, (KPW_12_/GO), TBA-PW_12_O_40_/GO, and a similar POM to the compound of interest, but Co-substituted Bp-PCoW_11_/GO. All the catalysts showed a gradual decrease in normalized substrate weight (*W*_*t*_/*W*_0_), confirming progressive starch conversion (Figure S9). However, it is worth noting that the GO-immobilized, metal-substituted catalysts (Bp-BCuW_11_/GO and Bp-BCoW_11_/GO) show the highest activity, with the Cu-substituted system achieving the fastest conversion. TBA-PW_12_/GO and KPW_12_/GO displayed moderate activity, confirming the beneficial role of GO in enhancing catalyst dispersion and substrate accessibility, though their catalytic performance remains inferior to that of the metal-substituted analogues. In contrast, unsupported KPW_12_/SWOH exhibits the lowest activity, highlighting the importance of immobilization and metal substitution in improving catalytic efficiency. Therefore, Bp-PW_11_Cu/GO still displayed a superior catalytic performance with a steady and gradual decrease over time.

The catalytic performance was further analyzed in terms of the reaction rate for starch hydrolysis to validate the trend observed in the experiment.^67,68^ As illustrated in Figure 4f, the reaction demonstrated first-order kinetics with respect to starch concentration, as substantiated by the linear representations of ln (*w*_*t*_/*w*_0_) versus hydrolysis time. The calculated rate constants (*k*), which are the slopes of the graphs, were 0.0628, 0.105, and 0.545, with linear regression coefficients R-squared of 0.939, 0.967, and 0.935, respectively, for the no-catalyst, Bp-PCuW_11_, and Bp-PCuW_11_/GO systems. Additionally, utilizing the Arrhenius equation to determine the activation energy (*E*_a_), the kinetic evaluation of the graph of ln *k* versus the reciprocal of Temperature (1/*T*), was performed as shown in Figure 4g. As illustrated in Figure 4h of the hydrolysis reaction, this revealed the apparent higher activation energies for no catalyst (110.5 kJ mol^–1^) and Bp-PCuW_11_ (57.9 kJ mol^–1^) than that obtained in Bp-PCuW_11_/GO (16.4 kJ mol^–1^).^69,70^ These kinetic parameters quantitatively confirm the enhanced catalytic activity of Bp-PCuW_11_/GO, exhibiting significantly higher hydrolysis rates. Overall, this substantial reduction in activation energy and enhancement in rate constant indicate that the catalyst alters the reaction pathway rather than merely supplying protons. If the process were purely acid-mediated, such pronounced kinetic differentiation would not be expected under identical acidity conditions.

#### 2.2.1 Optimization of the Catalyst

To elucidate the kinetics of the catalytic hydrolysis of starch facilitated by the Bp-PCuW_11_/GO catalyst, a comprehensive examination of various parameters was conducted. Besides the time of catalysis and mass ratio discussed earlier, other parameters, including starch concentration, dosage of Bp-PCuW_11_/GO, reaction temperature, reaction duration, and pH levels, were investigated. The influence of starch concentration on its catalytic hydrolysis was examined, with the catalyst quantity standardized to 20 wt % relative to the starch concentration, while the reaction temperature was maintained at 150 °C. The resulting products were analyzed at specified reaction intervals with starch concentrations adjusted to 10, 25, 50, 75, and 100 mg L^–1^. As shown in Figure 5a, at a starch concentration of 25 mg L^–1^, the glucose yield exhibited a gradual decline, culminating in the highest yield of 55% at the 2 h mark. Conversely, at a starch concentration of 50 mg L^–1^, the yield of monosaccharides progressively diminished from a peak yield of 65% at 1 h to a mere 52 wt % at 5 h. At starch concentrations of 75 or 100 g L^–1^, the starch substrate was not fully hydrolyzed at the initiation of the reaction, with the monosaccharide yield initially increasing before subsequently decreasing, achieving maximum yields of 63% and 59% at the 2 h interval, respectively. This observed initial augmentation in monosaccharide yield, followed by its decline, can be rationalized within the framework of a reaction sequence involving the acid-mediated hydrolysis of starch to monosaccharides, succeeded by their transformation into highly reactive C2 and C4 products via a retro-aldol reaction.^64^ Consequently, for the ensuing experimental investigations, the starch concentration was established at 50 g L^–1^.

**Figure 5:**
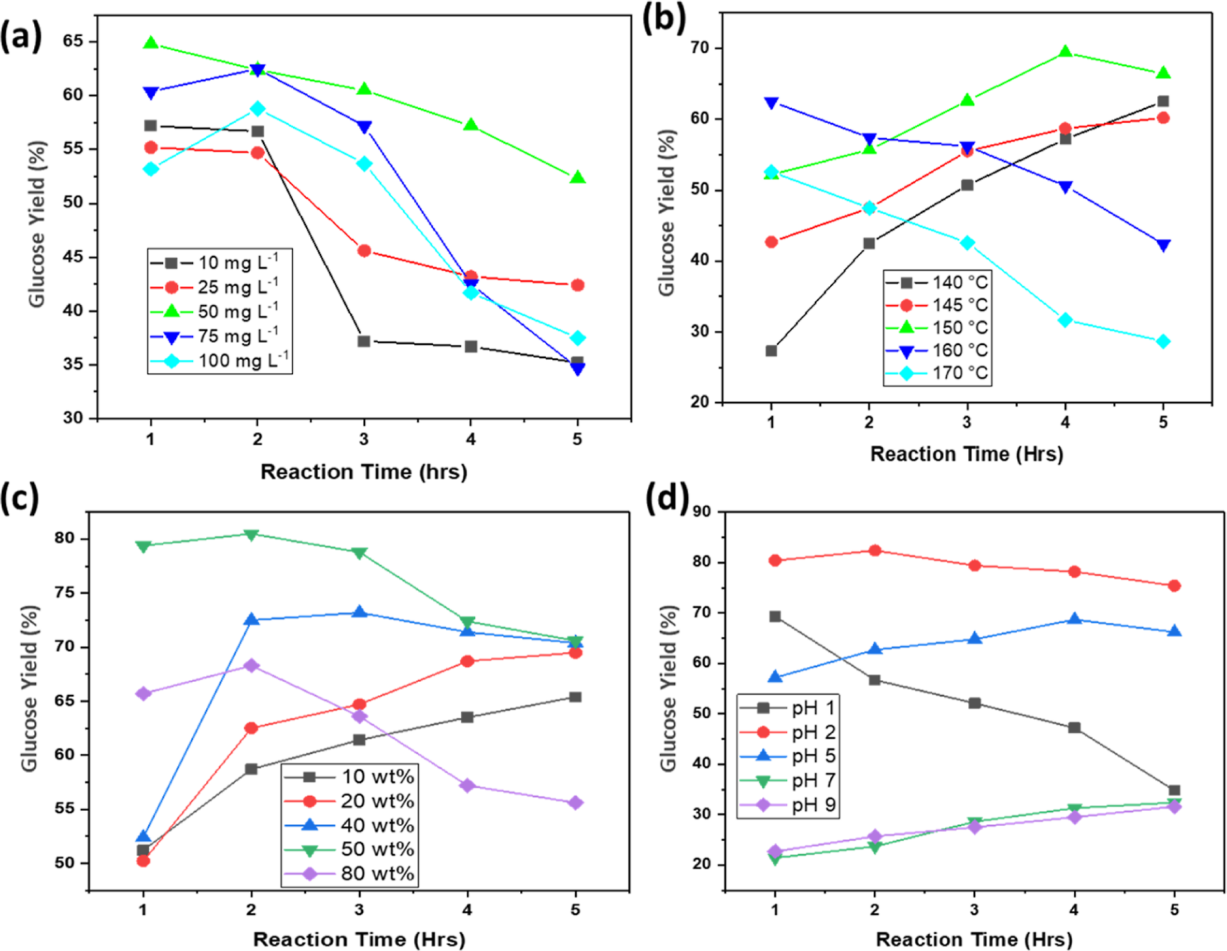
Effect on the starch hydrolysis: (a) concentration of Starch, (b) temperature, (c) amount of catalyst, (d) pH.

The temperature of the reaction system represents a pivotal determinant influencing the hydrolysis kinetics of starch. The catalytic efficacy of Bp-PCuW_11_/GO was systematically assessed at temperatures of 140, 145, 150, 160, and 170 °C, with a particular focus on the yield of monosaccharides. The evaluation of catalytic performance was conducted by standardizing the loading of Bp-PCuW_11_/GO catalyst at 20 wt %, the starch concentration at 50 mg L^–1^, and 5 h as the duration of the reaction. The findings are depicted in Figure 5b. At reaction temperatures of 140, 145, and 150 °C, the yield of monosaccharides exhibited an upward trend correlating with the extension of reaction time. Conversely, the yield of glucose displayed an initial increase followed by a decline, attaining a peak of 69 wt % at a temperature of 150 °C. At elevated reaction temperatures of 160 and 170 °C, the yield decreased with prolonged reaction time, attributed to the increased decomposition of monosaccharides under these thermal conditions. A significant cleavage of numerous C–O and C–C bonds within the molecular framework resulted in the generation of C2 and C4 small organic acids, while the condensation of monosaccharides gave rise to the formation of brown/black humins.^32,71^ The comparative analysis of the catalytic oxidation of starch across four distinct thermal conditions elucidates that at a reaction temperature of 150 °C, the yield of monosaccharides (65.2% at 240 min) was markedly elevated, thereby circumventing the inefficient hydrolysis of starch at higher temperatures. Consequently, 150 °C was identified as the optimal thermal condition for the reaction.

To investigate the influence of catalyst amount on the principal products arising from starch hydrolysis, the concentration of starch was maintained at 50 mg L^–1^, and the reaction temperature was established at 150 °C. As seen in Figure 5c, the yield of monosaccharides exhibited an upward trend corresponding to the prolongation of the reaction time when utilizing 10 wt % of Bp-PCuW_11_/GO, with the yield of monosaccharides reaching 69.4% after 5 h. When the amount of Bp-PCuW_11_ /GO was increased to 20 wt %, the yields of monosaccharides recorded were 70 wt % at 3 h. Furthermore, when the catalyst dosage was elevated to 40 wt %, the yields of monosaccharides attained 75% at the 2 h mark. Conversely, at a Bp-PCuW_11_/GO content of 50 wt %, analogous yields of monosaccharides were observed (80.7% at 2 h), which represented the maximum yield. The findings indicated that the yield was solely enhanced with an increase in reaction time, with the peak total yield of monosaccharides being 87% for a Bp-PCuW_11_/GO level of 20 wt %. It is noteworthy that an escalation in the catalyst dosage, from 40 to 50 and subsequently to 80 wt % of Bp-PCuW_11_/GO, resulted in only a marginal increase in yield to 72%, 81%, and 67%, respectively, yet a decline was observed with extended reaction time. Given that these variations were negligible, the optimal catalyst quantity was determined to be 20 wt % based on both economic considerations and theoretical data analysis.

In the context of acid hydrolysis of starch, pH emerges as a critical variable, given that the concentration of H^+^ ions significantly influences both the extent and kinetics of starch hydrolysis. The reaction medium was meticulously calibrated by adding 0.1 M HCl and 0.1 M NaOH incrementally to achieve the desired pH level. The yields of the main products of the Bp-PCuW_11_/GO-catalyzed starch hydrolysis at pH values of 1, 2, 5, 7, and 9 were investigated. As shown in Figure 5d, at a pH of 1, the concentration of H^+^ ions was significantly elevated within the reaction system, thereby promoting the complete acid hydrolysis of starch with a maximum yield of 69% of glucose. However, it progressively diminished with prolonged reaction time, ultimately dropping to 35% at 5 h. This could be attributed to the cascade of reaction degradation, which could lead to the formation of byproducts such as levulinic acid, glycolic acid, and humins.^72–74^ When the pH was 2, the yield of glucose exhibited an initial increase followed by a decline with extended reaction duration. Furthermore, the yield of glucose reached 82% at the 2 h interval. When the pH was 5, the absence of additional acid led to an increase in glucose yield, which subsequently decreased. Nonetheless, the peak yield of 69% was achieved at 4 h, occurring later than the peak at pH 2. At pH values of 7 and 9, the yields of monosaccharides increased correspondingly with the duration of the reaction. Nevertheless, after a reaction time of 4 h, the yields recorded were only 31% and 30%, respectively, which were considerably lower than those attained without the pH adjustment through the addition of NaOH. Consequently, it can be inferred that at a reduced pH (characterized by a high concentration of H^+^ ions), the hydrolysis of starch was markedly accelerated. The glucose yield at pH 2 was much higher than at all the other pH values analyzed, which was beneficial for producing glucose from starch. Overall, the optimized reaction conditions were as follows: the amount of the Bp-PCuW_11_/GO catalyst was set to 20 wt % of the starch concentration, the reaction system temperature was maintained at 150 °C, and the pH was adjusted to 2.

#### 2.2.2 Reusability of the Catalyst

The stability and recyclability of the catalysts are very important in practical applications. Therefore, the reusability of the Bp-PCuW_11_/GO catalyst was analyzed at optimized reaction conditions. After the reaction, unreacted starch and solid catalyst were separated via centrifugation. To test the residue in the mixture, the Bp-PCuW_11_/GO catalyst was extracted with diethyl ether, which was then filtered and dried. The solid catalyst was added to the freshly prepared starch for the next cycle, based on conversion, thereby serving as a representative candidate for assessing recycling efficacy. The results in Figure 6a indicate that glucose yields decreased slightly after ten runs, suggesting good recyclability of the Bp-PCuW_11_/GO catalyst. This also correlated with Figure 6b, which showed a reduction in catalyst activity, accompanied by a 3% loss in conversion efficiency after five runs. This may be attributed to the loss of experimental procedure efficiency due to the adsorption of many organics onto the catalyst.^63^ An outstanding catalytic activity was achieved, with glucose selectivity maintained at approximately 95%. The easy recovery and good reusability suggest a remarkable stability of the Bp-PCuW_11_/GO catalyst, showing negligible loss of activity in starch hydrolysis. Moreover, the ease of recovery via centrifugation and the preservation of catalytic efficacy across various applications render it a feasible substitute for practical implementations compared to volatile and corrosive liquid acids.^75,76^

**Figure 6:**
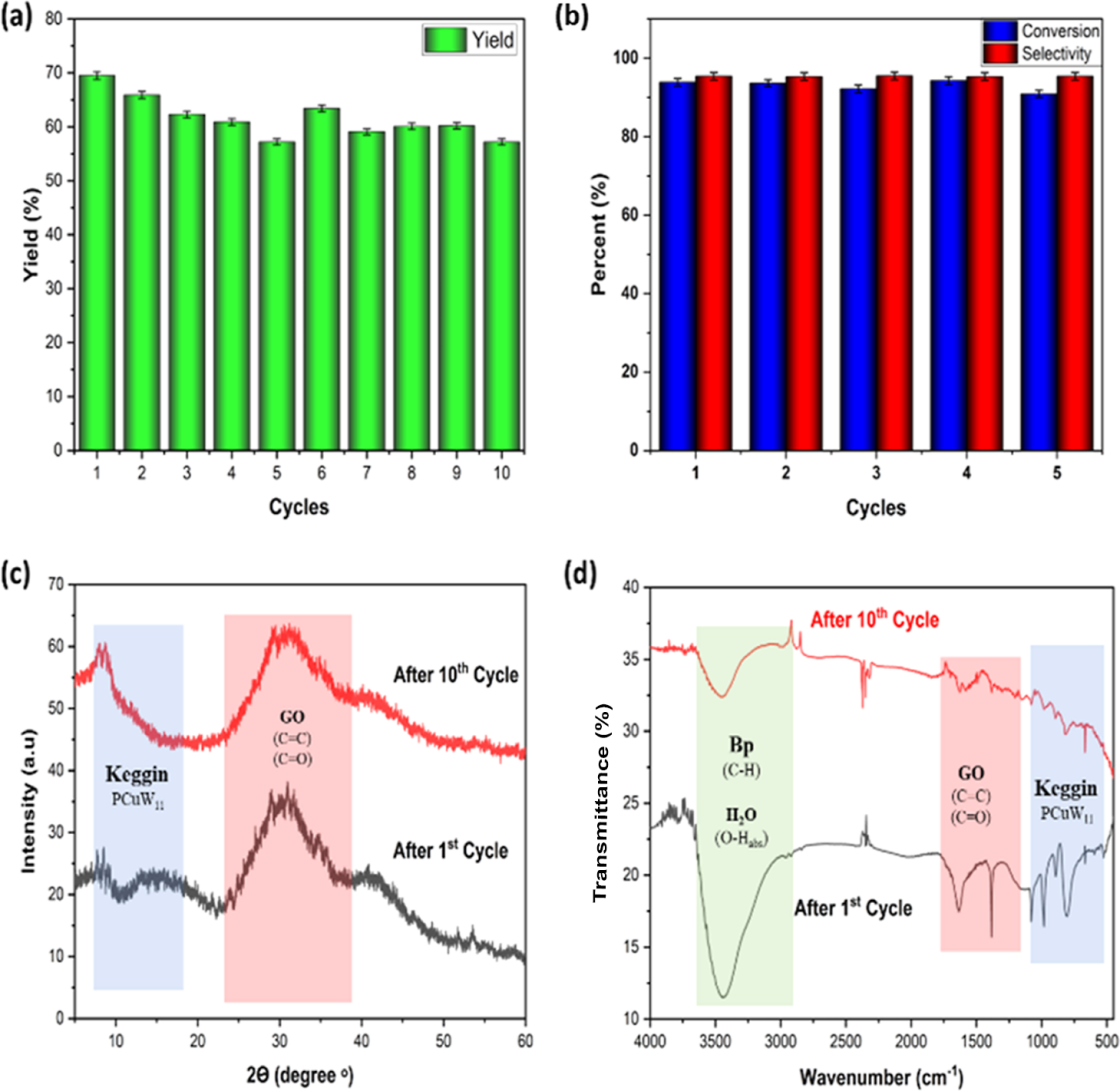
(a) Catalytic performance of the Bp-PCuW_11_/GO after 10 cycles. (b) The conversion efficiency and selectivity of Bp-PCuW_11_/GO nanohybrid catalysts after five cycles. Starch hydrolysis reaction after the first and tenth runs: (c) the XRD patterns (d) the FT-IR spectrum.

Acknowledging that a central driving force for the application of heterogeneous catalysts is the promotion of catalyst recovery, we performed an investigation into the features of the catalysts using powder XRD, FT-IR, and SEM analysis. The catalyst sample recovered after the first run and 10th cycle still shows well-maintained characteristic peaks from the Bp-PCuW_11_/GO catalyst sample, as shown by the XRD patterns in Figure 6c. Additionally, the FT-IR spectrum presented in Figure 6d exhibits strong, sharp peaks corresponding to the Bp-PCuW_11_/GO catalyst, further substantiating the maintenance of structural integrity after ten consecutive reaction cycles. Furthermore, the SEM imagery (Figure S10) elucidates that, despite retaining its spherical morphology, the spent catalyst exhibits an altered surface texture characterized by roughness, which can be attributed to the buildup of contaminants within the porous structures and on the surface of the catalyst.^77^ These studies suggested good stability under the optimized reaction conditions, as evidenced by the recovery of the catalyst from the hydrolysis of starch after ten cycles, underscoring the robustness of this catalytic system. Since many POMs are water-soluble and prone to leaching, successful reuse confirms that the POM remain firmly anchored on the GO support and retain their redox activity. This not only reduces material cost and environmental impact but also improves the practicality of the system for real catalytic applications. Consistent performance over multiple cycles further highlights the synergistic interaction between POMs and GO, making the hybrid more suitable for long-term and scalable use.

The Turnover-Frequency (TOF) = ([*w*_*t*_]/[mass of catalyst × time]) was determined to be 4.465 h^–1^ and 4.125 h^–1^ for Bp-PCuW_11_/GO and Bp-PCuW_11_, respectively. The Bp-PCuW_11_/GO exhibited higher TOF than Bp-PCuW_11_ did, which might be attributed to the interfacial catalysis and adsorption of some of the substrate to the GO. This enhancement arises from marked perfect synergistic interactions between the POM units and the GO framework. A comparison of the catalytic performance of Bp-PCuW_11_/GO with other reported POM-based heterogeneous solid acid catalysts for glucose generation is summarized in Table S3.^18,19,26,32,78–80^ A Bp-PCuW_11_/GO catalyst exhibits competitive or superior activity under comparable reaction conditions, offering the advantages of a mild operating temperature, high selectivity, and excellent recyclability. These results demonstrate the potential of POM–carbon hybrid materials as versatile and environmentally sustainable catalysts for biomass conversion applications.^32,81^

### 2.3 Computational Simulations and Mechanistic Analysis

DFT computations were employed to methodically examine the reaction mechanisms, intermediates, transition states, and energy barriers associated with the starch hydrolysis. Empirical evidence suggests that anchoring Bp-PCuW_11_ onto GO substantially enhances the catalytic activities and the yield obtained. This was demonstrated by examining the theoretical calculations of the catalyst surface’s electronic structure. Initially, we investigated the total density of states (TDOS), as shown in Figure 7a, which revealed a reduced band gap at the Fermi level. The anchoring of GO to Bp-PCuW_11_ shifted the Fermi level into the valence band, reducing the overall bandgap from 2.40 to 1.56 eV, which is conducive for electron transfer.^82^ Furthermore, we investigated the orbital coupling of the empty 3d orbitals of Cu in the Bp-PCuW_11_ framework and the 2p orbitals of O in H_2_O. When the H_2_O gets adsorbed on the Bp-PCuW_11_, the 2p orbitals of O overlap with the empty 3d orbitals of Cu, resulting in hydrogen transfer through the proton-accepting capability of the O active sites in the compound. As illustrated in Figure 7b, this interaction aligns well with the calculated HOMO–LUMO orbitals as verified by the ultraviolet photoelectron spectroscopy.^83^ The energy difference between the HOMO of H_2_O and the LUMO of PCuW_11_ for the Bp-PCuW_11_/GO (−4.2 eV) was lower than that for the pristine Bp-PCuW_11_ (−4.08). Therefore, this orbital interaction facilitates proton intermolecular transfer between the H_2_O and terminal O atoms (W=O_t_) in the POM, resulting in Lewis acidic characteristics. The protons can now be transferred to the electronegative oxygen atoms in the glycosidic bond in starch. This protonation of the glycosidic bond is identified as the rate-determining step. As demonstrated in Figure 7c, the Bp-PCuW_11_/GO nanohybrid (0.16 eV) exhibits a lower energy barrier than Bp-PCuW_11_ (0.23 eV), which is attributed to its increased surface area and active sites for protonation, favorable for starch hydrolysis. Thus, the presence of GO significantly enhances the acidity of the catalysts, thereby increasing the number of acidic sites. This is in agreement with the kinetic data, which show that the lower activation energy for catalytic activity was obtained for the Bp-PCuW_11_/GO nanohybrid. Additionally, the adsorption energies in Figure 7dshow that the presence of GO can significantly lower the H_2_O adsorption energy to −2.25 eV, compared to −0.68 eV for the GO-free nanohybrid. This is attributed to the more active O sites in GO, which effectively facilitate the activation of H_2_O adsorption.^84^ This was beneficial for the orbital interaction between Cu and O, as well as the subsequent protonation.

**Figure 7:**
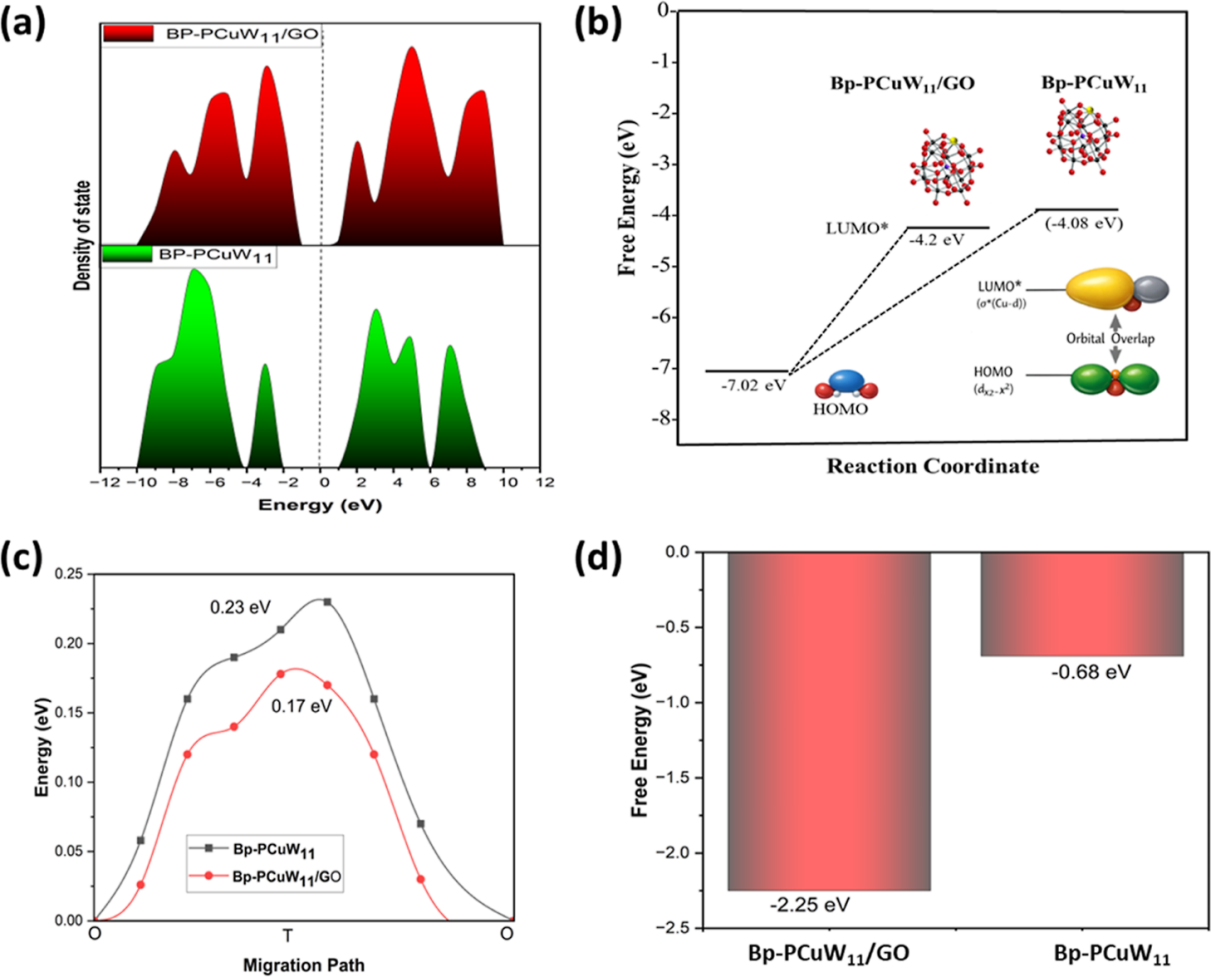
(a) TDOS spectra of Bp-PCuW_11_/GO and Bp-PCuW_11_ catalysts after H_2_O adsorption. (b) Orbital interaction mechanism between H_2_O and the Cu in the PCuW_11_ (c) energy barriers during the process of water adsorption. (d) The adsorption free energy diagrams for H_2_O on the Bp-PCuW_11_/GO and Bp- PCuW_11_ catalysts.

Based on the results of the above analysis, the plausible reaction mechanism of starch hydrolysis was proposed. As shown in Scheme 3, the Cu in PCuW_11_ cluster must first interact with H_2_O molecules to generate the active species with a metallic-oxo intermediate. Subsequently, the migration of H^+^ to the O in the glycosidic bond of starch is enantioselective, rather than the intermolecular migration to the W = O_t_ active species. The electrophilic C1 on the starch is now more susceptible to attack by OH^–^, which is then broken down to produce glucose. A similar mechanistic pathway is followed until a linear glucose chain is formed. This cooperative effect between Cu-substitution, the POM structure, and GO support represents a materials-driven catalytic enhancement, which differs fundamentally from conventional acid hydrolysis.

**Scheme 3:**
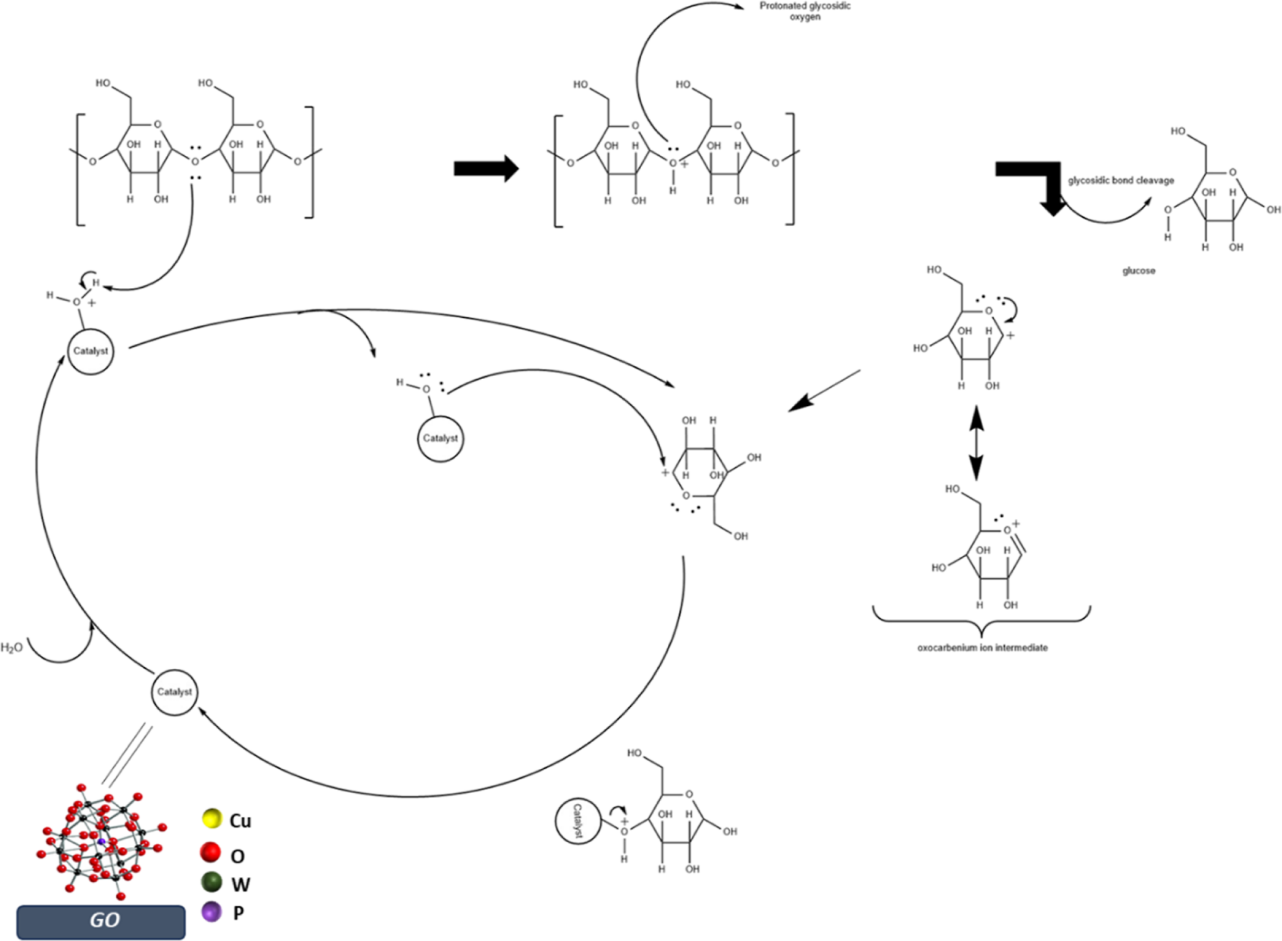
Plausible Mechanism for Catalytic Hydrolysis of Starch by Bp-PCuW_11_

## 3 Conclusion

In summary, the Bp-PCuW_11_/GO nanohybrid was successfully synthesized and demonstrated as an efficient and reusable solid catalyst for starch hydrolysis. The investigation of the catalytic activity of Bp-PCuW_11_/GO under hydrothermal conditions demonstrated improved performance compared to pristine Bp-PCuW_11_, attributed to the presence of GO, which increased the surface area, enhanced structural stability, reduced leaching tendency, and exposed active sites. The optimized reaction conditions were as follows: the amount of the Bp-PCuW_11_/GO catalyst was set to 20 wt % of the starch concentration, the reaction system temperature was maintained at 150 °C, and the pH was adjusted to 2. Under these optimized conditions, the PCuW_11_/GO catalyst achieved a 92% starch conversion, 82% glucose yield, and excellent selectivity of 95%, coupled with recyclability over five consecutive runs. The DFT simulation revealed that the superior catalytic efficiency is attributed to the synergistic effect between the Brønsted acidic Bp-PCuW_11_ units and the hydrophilic, high-surface-area GO support, which collectively enhances proton transfer, substrate adsorption, and mass transport during the hydrolysis process. This work demonstrates that rational integration of POMs with graphene-based support can yield high-performance solid acid catalysts with tunable acidity, stability, and selectivity. Furthermore, the nanohybrids based on POM and nanocarbon systems offer a sustainable platform for biomass valorization and can be extended to other acid-catalyzed transformations, such as cellulose hydrolysis, esterification, and oxidative reactions in green chemistry applications.

## 4 Methods

### 4.1 Materials and Reagents

All chemicals were purchased from commercial sources and were used as received without further purification. These are CuCl_2_·2H_2_O (Merck ⩾ 99%), Co­(NO_3_), 6H_2_O (Merck ⩾ 99%), Na_6_[H_2_W_12_O_40_] (Alfa Easer 99%), NaNO_3_ (Merck ⩾ 99%), H_3_PO_4_ (Sigma-Aldrich, 85%), KMnO_4_ (Merck ⩾ 99%), 4,4′-bipyridine (Alfa Aesar 98%), H_2_SO_4_ (Merck, 98%), H_2_O_2_ (Sigma-Aldrich, 30%) and Graphite powder (Nanoka, ≥99%), Natural starch (analytical grade) was purchased from Merck. Deionized water was used throughout all experiments.

### 4.2 Catalyst Preparation

#### 4.2.1 Preparation of Bp-CuPW_11_

In the synthesis of Bp-PCuW_11_, the literature procedure was followed,^34^ which involved a mixture of Na_6_[H_2_W_12_O_40_] (0.1 mmol), CuCl_2_·2H_2_O (0.5 mmol), 4,4′-bipyridine (0.5 mmol), and H_3_PO_4_ (0.3 mmol). The resultant mixture was stirred for 1 h and then transferred into a Teflon-lined autoclave (23 mL internal volume), which was heated at 170 °C for 3 days. Following the cooling process to ambient temperature, the resultant products, which comprised, a mixture of pale blue columnar crystals and blue powder, were subjected to filtration and subsequently washed with distilled water and acetone. The crystalline products were carefully isolated in mineral oil manually under an optical microscope. Only the pale blue columnar crystals were selected for further characterization and subsequent experiments. The phase purity and identity of the isolated compound as [(4,4′-bpyH_2_)_2_(4,4′-bpyH)]­[PCuW_11_O_39_]·H_2_O (Bp-PCuW_11_) were confirmed by PXRD, FT-IR, XPS, and EDX elemental composition analysis. The other visually distinct crystalline forms were not included in this study.

#### 4.2.2 Synthesis of GO

GO was prepared from natural graphite powder via a modified Hummers’ method.^35,36^ Briefly, graphite powder (2.12 g) and NaNO_3_ (1.01 g) were added to 50 mL of concentrated H_2_SO_4_ (98%) while stirring in an ice bath. Then, KMnO_4_ (6 g) was slowly added, ensuring the temperature remained below 10 °C. The resultant mixture was agitated for 2 h and then gradually heated to 35 °C for an additional 2 h. Subsequently, 100 mL of deionized water was added slowly, followed by heating to 90 °C for 30 min. The reaction was ultimately halted by the addition of 10 mL of 30% H_2_O_2_, yielding a bright yellow suspension. The obtained GO was subjected to washing with 1.0 M HCl and deionized water until the pH reached neutrality, then dried at 60 °C for 24 h.

#### 4.2.3 Synthesis of Bp-CuPW_11_/GO

The preparation of the Bp-PCuW_11_/GO hybrid was carried out using a Retsch PM 100 planetary ball mill. Initially, 0.25 g of GO and 5 g of Bp-PCuW_11_ were premixed using a mixer grinder to ensure homogeneous blending. The resulting composite mixture was then transferred into a one-liter milling cylinder equipped with a high-density polyethylene (HDPE, Nalgene) jar. Yttria-stabilized zirconia balls (5 mm diameter, 0.4 g per ball) were used as the grinding media, maintaining a ball-to-powder ratio of 15:1. Milling was performed at a rotational speed of 80–120 rpm for 8 h. To prevent overheating during prolonged milling, the process was conducted with intermittent pauses. A similar procedure, is in the Supporting Information, was employed to prepare nanohybrids for other heterogeneous catalysts, K_3_PW_12_W_40_/SWCNT (KPW_12_/SWOH), K_2_PW_12_W_40_/GO, (KPW_12_/GO), TBA-PW_12_O_40_/GO, and a similar POM to the compound of interest, but Co-substituted Bp-PCoW_11_/GO. Their catalytic performance was examined in the hydrolysis of starch.

### 4.3 Catalysis of Starch

The catalytic reaction was conducted in a 25 mL stainless steel autoclave. A typical catalytic reaction procedure was as follows: starch (0.1 g), catalyst (100 mg), and distilled water (7 mL) were mixed by magnetic stirring (30 rpm). The mixture was introduced into a steel autoclave lined with Teflon in an atmospheric environment, and the autoclave was subjected to a thermal treatment at 150 °C for 5 h. The reaction was terminated by swiftly cooling the reactor in an ice bath maintained at 0 °C. The catalyst was separated by centrifuge at 5000 rpm for 10 min and filtered. The filtrate was tested for the presence of starch using the Lugol iodine test and analyzed for glucose concentration using a UV–Vis spectrophotometer (λ = 505 nm) via the DNS (3,5-dinitrosalicylic acid) method.^62^ Control experiments using pristine GO and pure Bp-CuPW_11_ were conducted for comparison. The reusability of the catalyst was examined over ten consecutive runs by washing the recovered Bp-CuPW_11_/GO with water and drying it after each cycle.

The catalytic performance metrics were evaluated in terms of conversion, yield of glucose, and selectivity, defined as follows

1 $$convesion\left(\%\right)=\left(1-\frac{W_{t}}{W_{0}}\right)\times 100$$

2 $$yield\left(\%\right)=\frac{W_{t}}{W_{0}}\times 100$$

3 $$selectivity\left(\%\right)=\frac{yield}{conversion}\times 100$$

where *W*_0_, and *W*_*t*_ denote the initial starch concentration, and the residual starch concentration after time *t*, respectively.

### 4.4 Material Characterization

Scanning electron microscopy (SEM) analyses were conducted to investigate the morphological characteristics, dimensions, and microstructural properties of the materials under examination. Powder X-ray diffraction (XRD) patterns were acquired utilizing a Rigaku Smart Lab SE apparatus, operated at 30 kV and 20 mA, employing Cu Kα radiation (λ = 0.15406 nm), across a 2θ range extending from 10° to 80°, thereby facilitating the identification of the powdered phase of the materials. The vibrational peaks were recorded using a Thermal Scientific Fourier Transform Infrared (FT-IR) spectrometer by combining the samples with KBr pellets. Thermogravimetric analysis (TGA, STA-449-F3) was performed in an air environment, using a heating rate of 10 °C min^–1^ across a temperature range from ambient temperature to 800 °C. Ultraviolet–visible (UV–Vis) absorption spectra for all samples were recorded using a Lambda 750 spectrophotometer (PerkinElmer). The NMR spectra of the compounds were measured on an Agilent 600 MHz Nuclear Magnetic Resonance Spectrometer, using D_2_O as the solvent and tetramethylsilane (TMS) as the internal standard. The quantification of specific surface area and pore architecture was performed utilizing the Brunauer–Emmett–Teller (BET) methodology at 77 K, employing apparatus from Micromeritics Instrument Corporation. X-ray photoelectron spectroscopy (XPS) for the analysis of chemical composition and surface electronic states was performed using a PHOIBOS HSA3500 DLD 150 R6-HiRes [HW Type 30:101] DLD, which utilized a nonmonochromatic Mg Kα radiation X-ray source.

### 4.5 Computational Analysis

All computational analyses were conducted within the framework of the generalized gradient approximation (GGA), utilizing the Perdew–Burke–Ernzerhof (PBE) functional within the Vienna Ab Initio Simulation Package (VASP), which employs the plane-wave-based periodic density functional theory (DFT) methodology.^85^ At the same time, the core electrons of the respective elements were addressed through the projected augmented wave (PAW) configurations. The energy cutoff was set at 450 eV, and a 3 × 3 × 1 Monkhorst–Pack *k*-point grid was used. All surface structures were constructed using periodic slabs comprising four atomic layers, wherein the bottom two layers of atoms were constrained. The forces acting on the atoms were optimized to remain below 0.01 eV Å^–1^. The transition states (TSs) were identified using the climbing image nudged elastic band (CI-NEB) technique and corroborated by the presence of a single imaginary vibrational mode along the reaction coordinate.

## Supplementary Material



## Abbreviations

POM: polyoxometalates
GO: graphene oxide
XRD: X-ray diffraction
DFT: density functional theory.
