# Supplementary material for: Immobilized Copper-Substituted Keggin POM on Graphene Oxide for Highly Selective Heterogeneous Catalysis of Starch Hydrolysis
Source: ACS Omega. 2026 Jun 3;11(23):33832–48. doi: 10.1021/acsomega.6c00419 (PMC13280877; doi:10.1021/acsomega.6c00419)
Supplement: Supplementary file 1 [file ao6c00419_si_001.pdf]

Supporting Information

Immobilized Copper-substituted Keggin POM on Graphene Oxide for Highly Selective Heterogeneous Catalysis of Starch Hydrolysis

Langson Chilufya[1,2], Bright Chilikwazi[2], Beraat Umur Kaya[1] and Mehtap Emirdag-Eanes*[1]

[1]*Department of Chemistry, Faculty of Science, Izmir Institute of Technology, Gülbahçe Campus 35430 Urla, İzmir, 35050, Türkiye.*

[2]*Department of Pure and Applied Chemistry, School of Natural Sciences, University of Zambia, P. O. Box 32379, Lusaka, 10101, Zambia.*

Content

1. **Experimental**
   1.1. **Synthsis of $(4,4'\text{-bpyH}_2)_2(4,4'\text{-bpyH})[PCoW_{11}O_{39}]\cdot H_2O$ (Bp-$PCoW_{11}$)**
   1.2. **Synthesis of $K_3[PW_{12}O4_0]$. xH_2O (KPW_{12}) and $\{(C_4H_9)_4N\}$ $[PW_{12}O4_0]$. xH_2O (TBA-PW_{12})**
2. **Figures and Tables**
   - **Figure S1**. a) The generated PXRD of Bp-PCuW11 single crystal X-ray results using Mercury program and experimental PXRD of Bp-PCuW11. b) Crystal pictures of Bp-$PCuW_{11}$.
   - **Figure S2**. The XRD spectra for comparison of the ball milled prepared Bp-$PCuW_{11}$/GO and the manually mixed Bp-$PCuW_{11}$ + GO
   - **Figure S3**. The FT-IR spectra for comparison of the ball milled prepared Bp-PCuW11/GO and the manually mixed Bp-$PCuW_{11}$ + GO
   - **Figure S4.** The image of Bp-$PCuW_{11}$ in analyzed by (a) SEM at 1 µm and (b) STEM at 300 nm
   - **Table S1.** Comparative analysis of the Brunauer-Emmett-Teller (BET) of the materials
   - **Figure S5**. The XPS data of Bp-$PCuW_{11}$: (a) Survey (b) W 4f (c) Cu 2p (d) C 1s (e) N 1s (f) O 1s
   - **Table S2**. Comparative analysis XPS binding energies of the Bp-$PCuW_{11}$ and Bp-$PCuW_{11}$/GO materials
   - **Figure S6**. The mass ratio of GO to Bp-$PCuW_{11}$, mixed in ball milling, corresponds to 1:20, with 0.25g of GO and 5.0g of Bp-$PCuW_{11}$, yielding a maximum of 90%.
   - **Figure S7**. The varying color intensity with time following the DNS method for reducing sugars in the hydrolysis of starch.
   - **Figure S8**. Hot filtration experiment of Bp-$PCuW_{11}$/GO
   - **Figure S9**. The heterogeneous catalysts examined in starch hydrolysis.
   - **Figure S10.** The SEM images after starch hydrolysis for the (a) first and (b) tenth run.
   - **Table S3**. Comparison of the catalytic performance with previously reported POM materials for glucose production.

1. Experimental

1.1. Synthsis of $(4,4'\text{-bpyH}_2)_2(4,4'\text{-bpyH})[PCoW_{11}O_{39}]\cdot H_2O$ (Bp-$PCoW_{11}$)

Literature procedure was used to prepare Bp-$PCoW_{11}$.[1] Briefly, the reaction mixture was prepared by stirring $Na_2WO_4.2H_2O$ (0.3023 g), $Co(NO_3)_2.6H_2O$ (0.2912 g), 4,4'-bipyridine (0.1084 g), 5M H_3PO_4 (85%, 0.5 mL) in 7.0 mL of H_2O for 10 minutes. This reaction mixture was transferred to the Teflon vessel line autoclave (23 mL internal volume). This was placed in an oven and heated at 170°C for 3 days. After cooling to room temperature, the mixture was filtered and washed three times with distilled water. A mixture of red and colourless crystals was then observed, which were separated under a microscope. Red crystals were a well-known compound **Bp-PCoW$_{11}$**.

1.2. Synthesis of $K_3[PW_{12}O4_0]$. xH_2O (KPW_{12}) and $\{(C_4H_9)_4N\}$ $[PW_{12}O4_0]$. xH_2O (TBA-PW_{12})

The other POMs examined in the starch hydrolysis, KPW_{12} and TBA-PW_{12}, were prepared as described in our previous works.[2] Briefly, for the preparation of $KPW_{12,}$ an excess aqueous solution of KCl (100 mL, 0.21 mol/L) was added dropwise to an aqueous solution of PW_{12} (200 mL, 0.034 mol/L) at room temperature with vigorous stirring for 10 hours. The precipitate was centrifuged at 3000 rpm for 30 min, washed thoroughly with water, and dried at 80 °C in an oven. Similarly, TBA-PW_{12} was prepared using this procedure, with the only change being the substitution of TBA-Cl (20 mL of 0.072 M) for KCl.

2. Supplementary Figures and Tables

a)

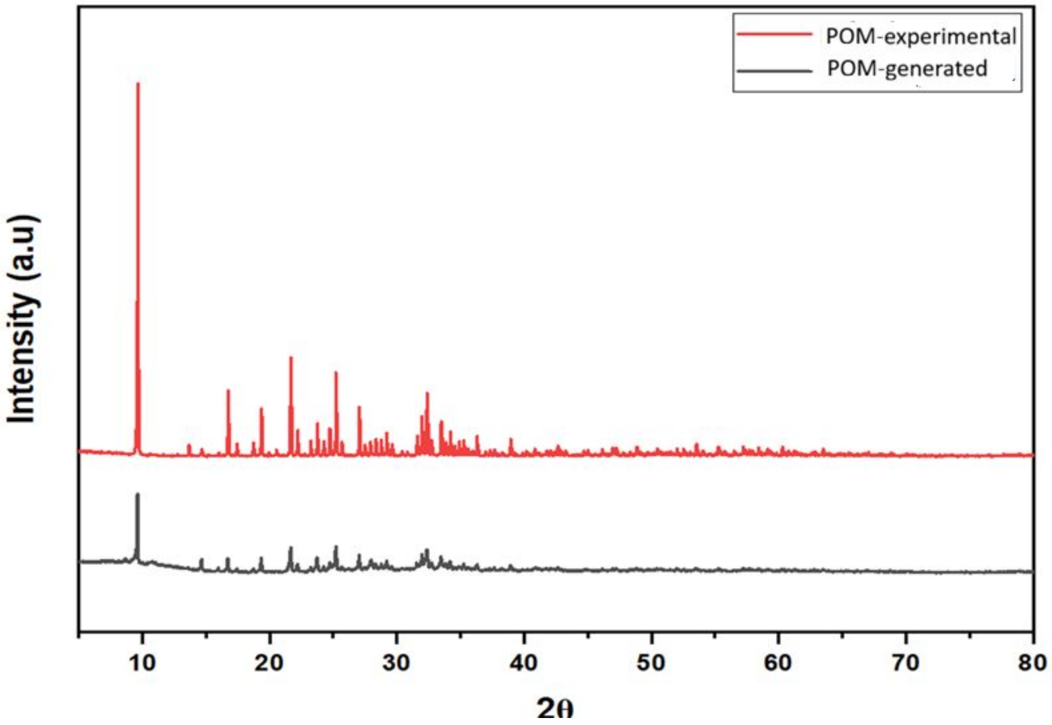

b)

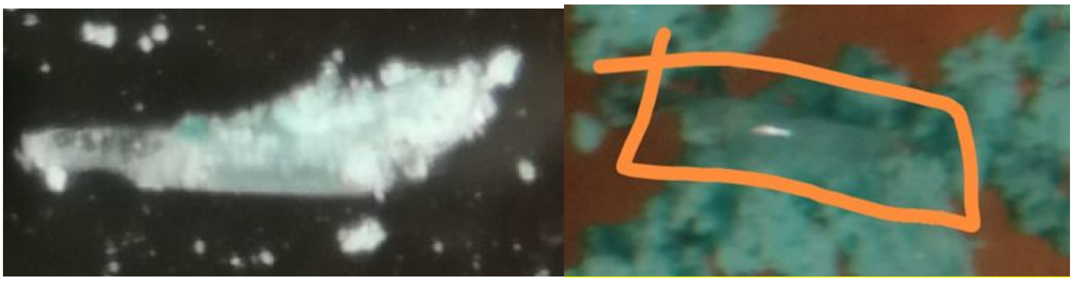

Figure S1. a) The generated PXRD of Bp-PCuW$_{11}$ single crystal X-ray results using Mercury program and experimental PXRD of Bp-PCuW$_{11}$ b) Crystal pictures of Bp-PCuW$_{11}$.

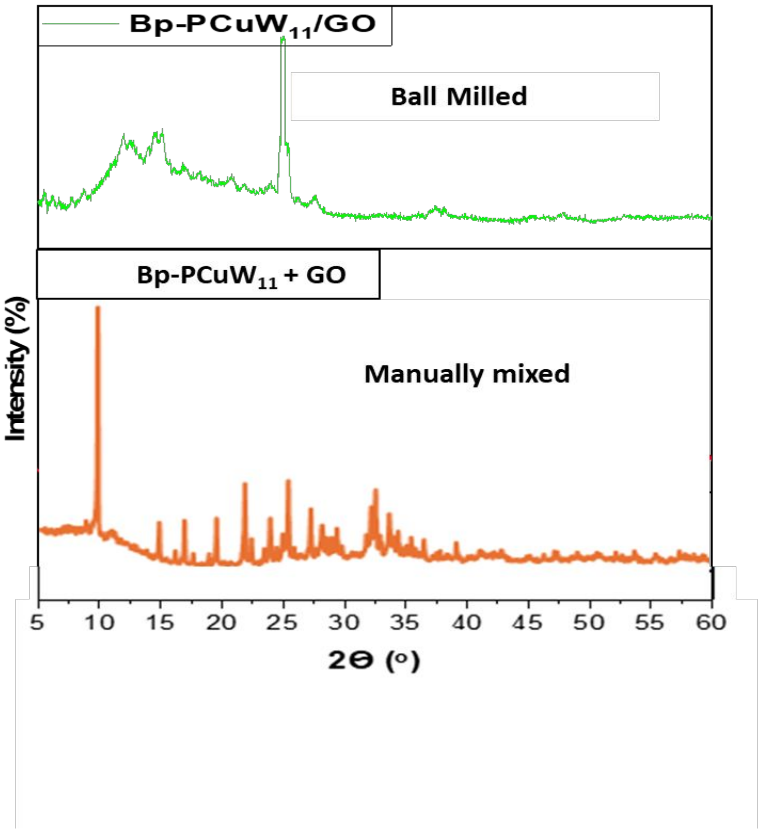

Figure S2. The XRD spectra for comparison of the ball milled prepared Bp-$PCuW_{11}$/GO and the manually mixed Bp-$PCuW_{11}$ + GO.

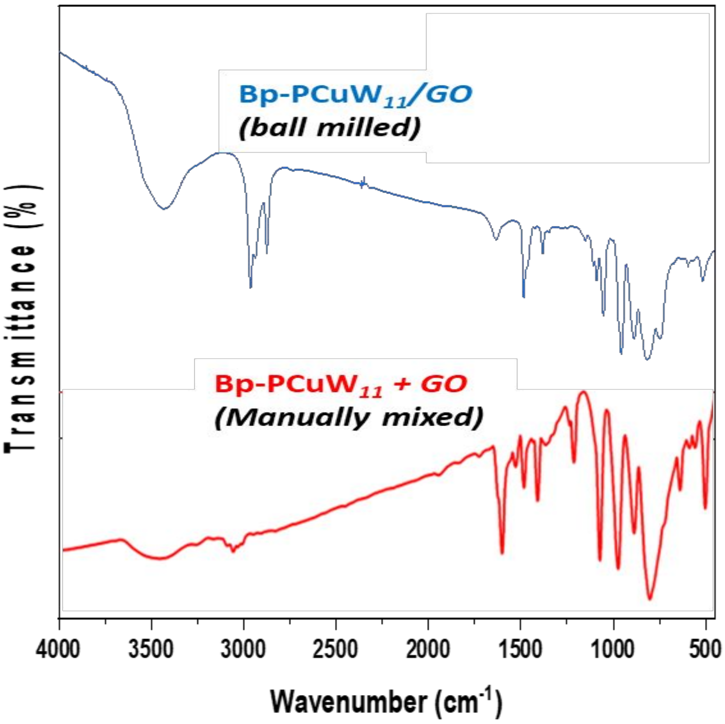

Figure S3. The FT-IR spectra for comparison of the ball milled prepared Bp-PCuW$_{11}$/GO and the manually mixed Bp-PCuW$_{11}$ + GO.

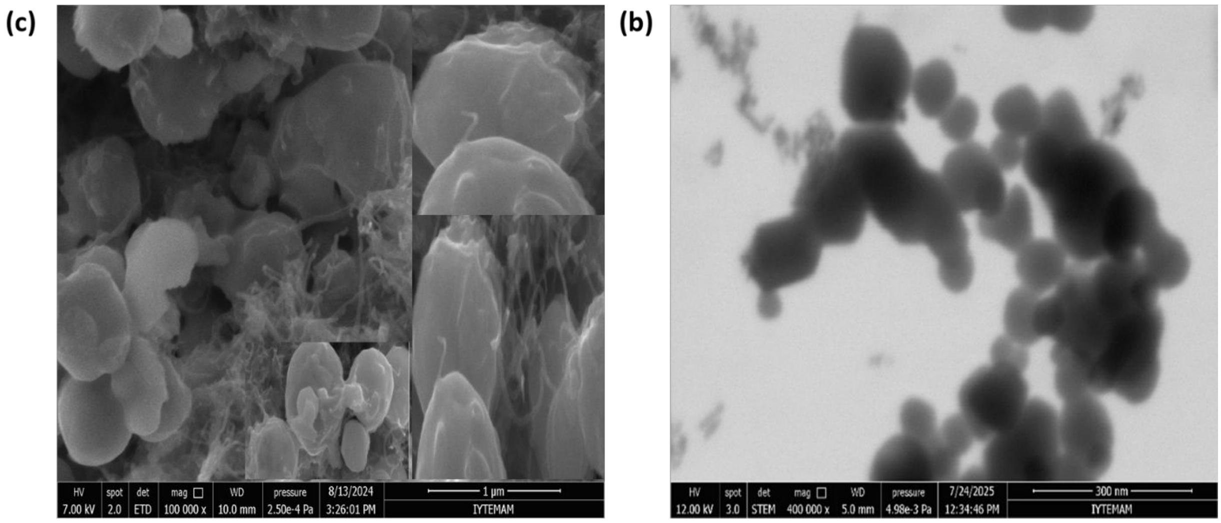

Figure S4. The image of Bp-PCuW$_{11}$ in analyzed by (a) SEM at 1 μm and (b) STEM at 300 nm

Table S1. Comparative analysis of the Brunauer-Emmett-Teller (BET) of the materials

Compound	BET Surface Area (m^2g^{-1})	Pore Size (nm)	Micropore Volume (cm^3g^{-1})
Bp-$PCuW_{11}$/GO	127.6	2.7	0.25
Bp-$PCuW_{11}$	87.4	1.8	0.16

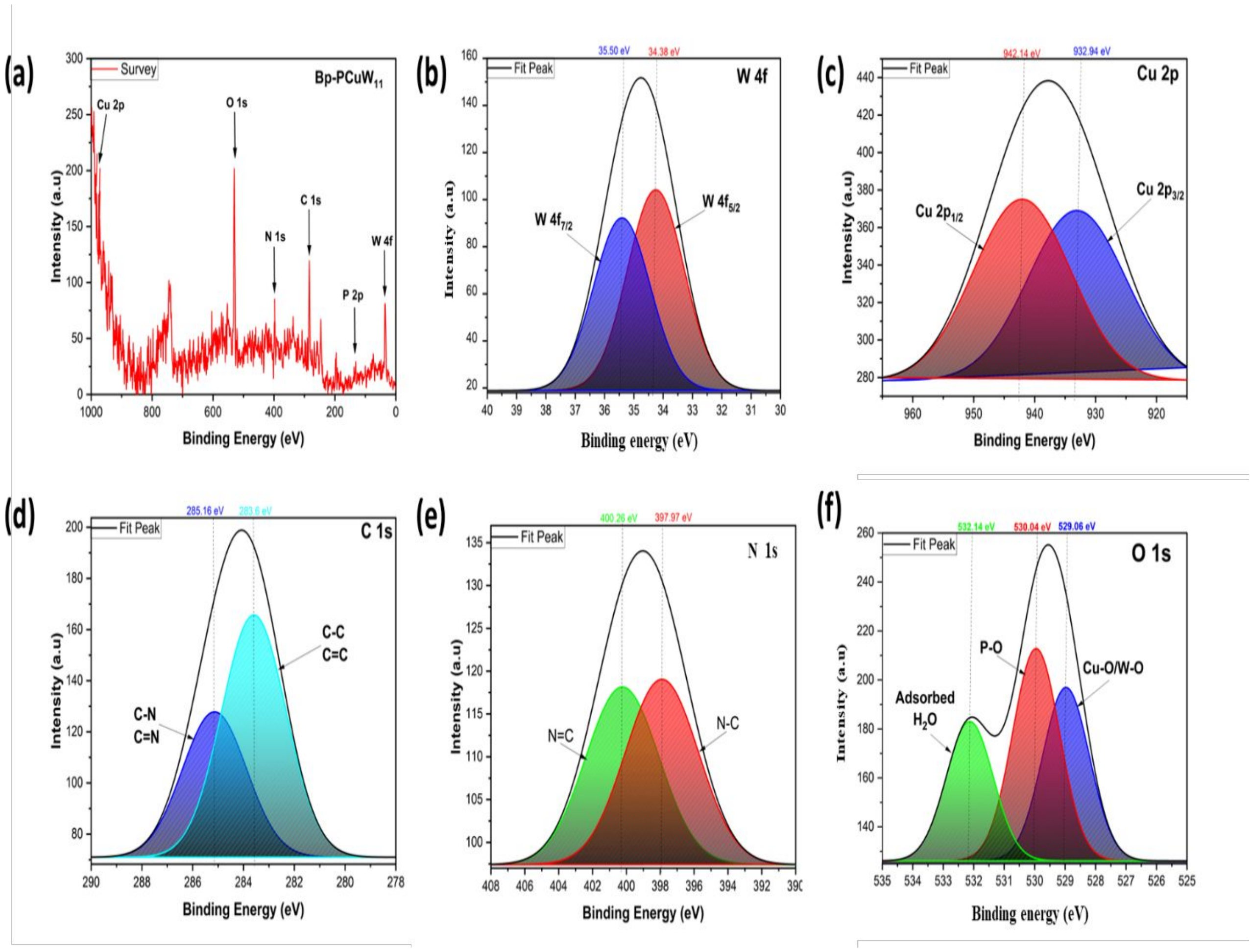

Figure S5. The XPS data of Bp-$PCuW_{11}$: (a) Survey (b) W 4f (c) Cu 2p (d) C 1s (e) N 1s (f) O 1s

Table S2. Comparative analysis XPS binding energies of the Bp-$PCuW_{11}$ and Bp-$PCuW_{11}$/GO materials

	Oxidation State or Bonding	Bp-$PCuW_{11}$	Bp-$PCuW_{11}$/GO
**W 4f**	W $4f_{7/2}$	35.5	36.44
	W $4f_{5/2}$	34.38	34.64
**Cu $2p_{3/2}$**	Cu^{+}	932.94	934.16
	Cu^{2+}	939.53	934.41
**Sat.**		943.34	942.51
**Cu$2p_{1/2}$**	Cu^{+}	942.14	946.74
	Cu^{2+}	954.21	953.82
**Sat.**		962.51	962.82
**O1s**	O-H_{abs}	532.14	531.68
	W-O/Cu-O	529.06	528.92
	P-O	530.04	529.76
	C-O	-	530.67
	C=0	-	532.88
**N1s**	N-C	397.97	398.39
	N=C	400.26	400.39

**C1s**	C-C/C=C	283.60	282.81
	C-O	-	283.76
	C=O	-	284.47
	C-N/C=N	285.16	285.01

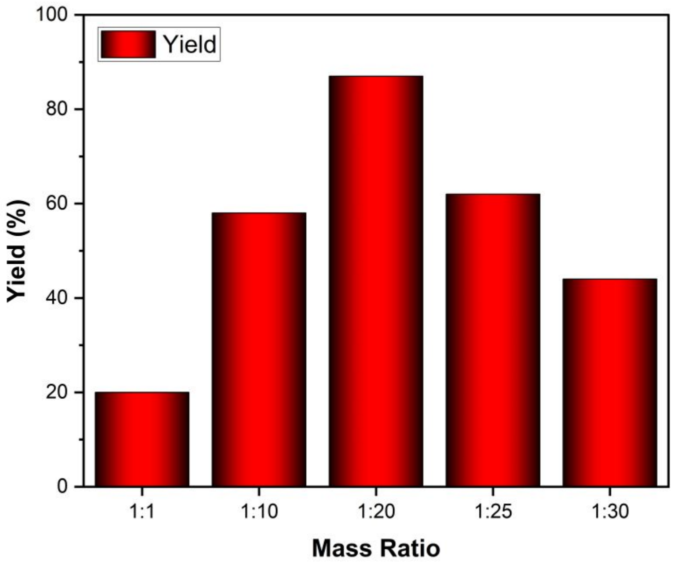

Figure S6. The mass ratio of GO to Bp-PCuW_{11}, mixed in ball milling, corresponds to 1:20, with 0.25g of GO and 5.0g of Bp-PCuW_{11}, yielding a maximum of 90%.

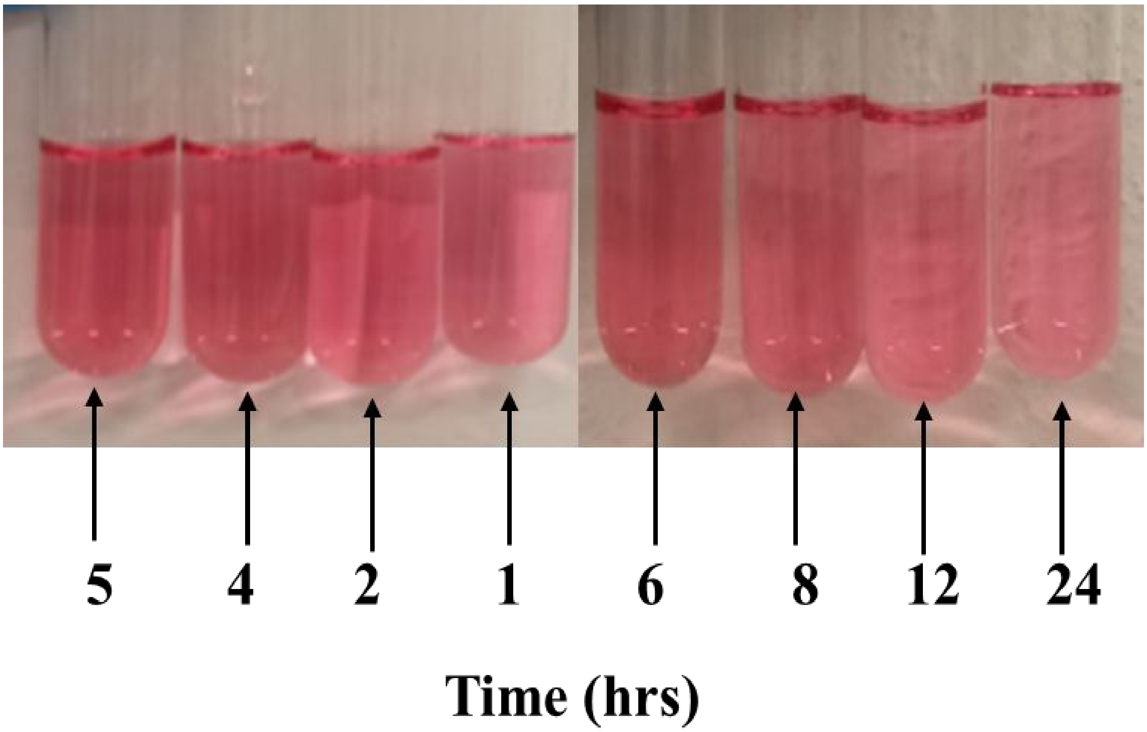

Figure S7. The varying color intensity with time following the DNS method for reducing sugars in the hydrolysis of starch.

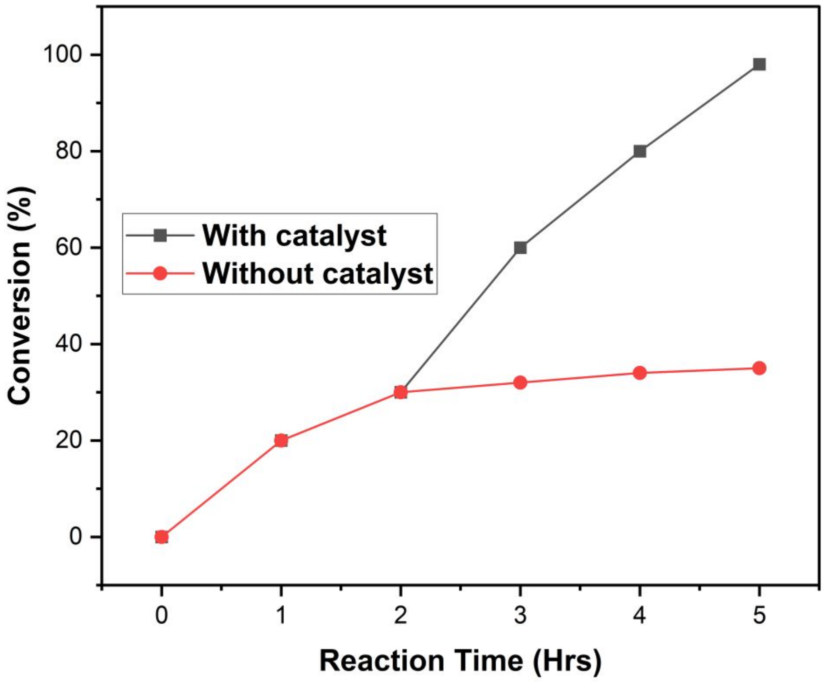

Figure S8. Hot filtration experiment of Bp-$PCuW_{11}$/GO

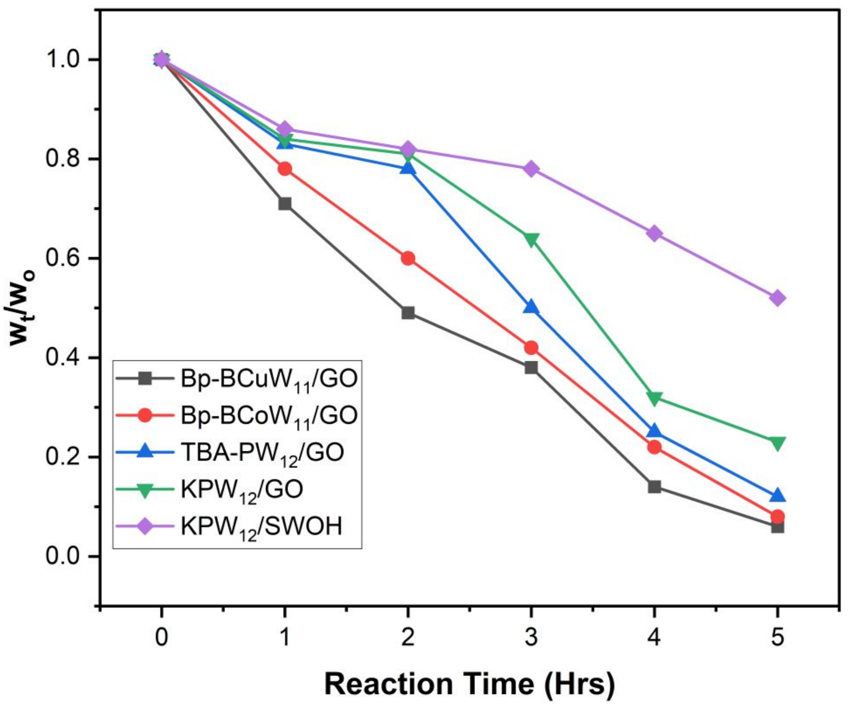

Figure S9. The heterogeneous catalysts examined in starch hydrolysis.

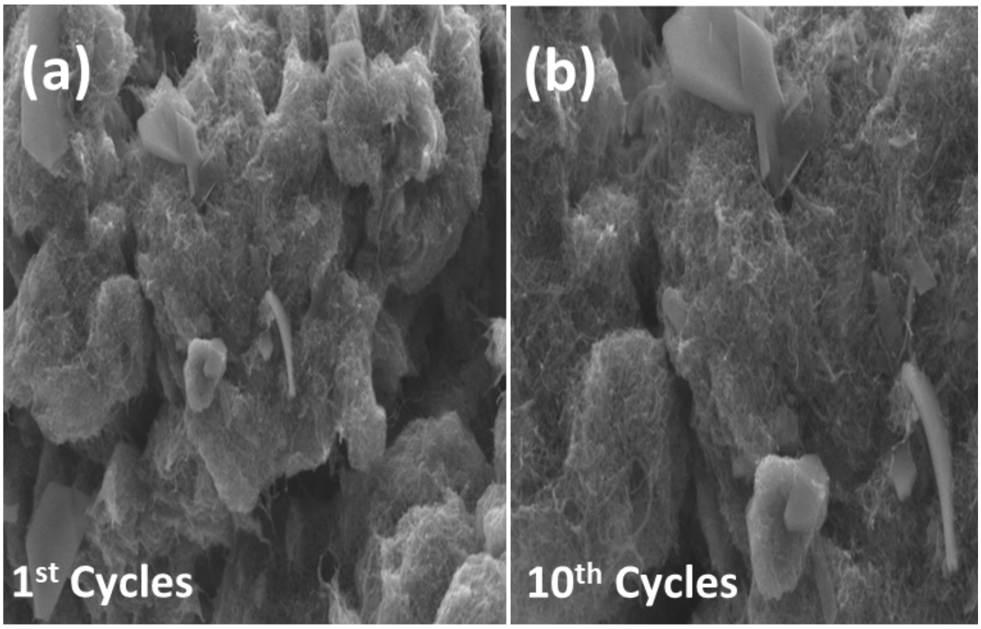

Figure S10. The SEM images after starch hydrolysis for the (a) first and (b) tenth run.

Table S3. Comparison of the catalytic performance with previously reported POM materials for glucose production

Compound	Substrate	Conversion (%)	Selectivity (%)	Cycles	TOF (h^{-1})	Ref
Bp-$PCuW_{11}$/GO	Starch	92	94.8	10	4.47	This work
$H_3PW_{12}O_{40}nH_2O$	Glycogen	87	89	2	-	3
(4,4′-bpyH_2)$_3$[$PW_{12}O_{40}$]$_2$·3H_2O	Starch	90	-	5	-	4
$H_3[PW_{12}O_{40}]$	Cellulose	90.1	86	-	-	5
$H_3[PW_{12}O_{40}]$	Starch	-	90	4	-	6
$[C_{16}H_{33}N(CH_3)_3]H_2PW_{12}O_{40}$	Starch	94	85.7	5	-	7
$HSiW_{12}$/G	Glycogen	66	86	3	-	8
HPW_{12}/g-C_3N_4	Cellulose	-	81.6	10	2.56	9

3. References.

(1) Wang, J. P.; Shen, Y.; Niu, J. Synthesis, Characterization and Crystal Structure of a 1D Heteropolytungstate [4,4′-BipyH2]2[4,4′-BipyH] [PCoW11O39]·H2O. *J Coord Chem* 2007, *60* (11), 1183–1190. https://doi.org/10.1080/00958970601026699.

(2) Chilufya, L.; Sertbaş, V.; Aytekin, A.; Karabudak, E.; Emirdag-Eanes, M. Investigation on the Keggin Anchored on Hydroxide-Functionalized Single-Walled Carbon Nanotubes as Superior Cathode for Aqueous Zinc-Ion Batteries. *ACS Omega* 2025, *10* (32), 36536–36549. https://doi.org/10.1021/acsomega.5c05213.

(3) Klein, M.; Pulidindi, I. N.; Perkas, N.; Gedanken, A. Heteropoly Acid Catalyzed Hydrolysis of Glycogen to Glucose. *Biomass Bioenergy* 2015, *76*, 61–68. https://doi.org/10.1016/j.biombioe.2015.02.036.

(4) Ece, Ö.; Chilufya, L.; McMillen, C. D.; Emirdag-Eanes, M. Hydrothermal Synthesis, Characterization and Catalytic Activities of a Keggin Structure of (4,4′-BpyH2)3[PW12O40]2·3H2O and a Wells-Dawson Structure of (4,4′-BpyH2)3(4,4′-BpyH)1.75[Cu(Bpy)2]0.25[H2P2W18O62]2. *J Mol Struct* 2025, *1322*. https://doi.org/10.1016/j.molstruc.2024.140556.

(5) Nakamura, M.; Islam, M. S.; Rahman, M. A.; Nahar, R. N.; Fukuda, M.; Sekine, Y.; Beltramini, J. N.; Kim, Y.; Hayami, S. Microwave Aided Conversion of Cellulose to Glucose Using Polyoxometalate as Catalyst. *RSC Adv* 2021, *11* (55), 34558–34563. https://doi.org/10.1039/d1ra04426e.

(6) Tsubaki, S.; Oono, K.; Ueda, T.; Onda, A.; Yanagisawa, K.; Mitani, T.; Azuma, J. I. Microwave-Assisted Hydrolysis of Polysaccharides over Polyoxometalate Clusters. *Bioresour Technol* 2013, *144*, 67–73. https://doi.org/10.1016/j.biortech.2013.06.092.

(7) Cheng, M.; Shi, T.; Guan, H.; Wang, S.; Wang, X.; Jiang, Z. Clean Production of Glucose from Polysaccharides Using a Micellar Heteropolyacid as a Heterogeneous Catalyst. *Appl Catal B* 2011, *107* (1–2), 104–109. https://doi.org/10.1016/j.apcatb.2011.07.002.

(8) Klein, M.; Varvak, A.; Segal, E.; Markovsky, B.; Pulidindi, I. N.; Perkas, N.; Gedanken, A. Sonochemical Synthesis of HSiW/Graphene Catalysts for Enhanced Biomass Hydrolysis. *Green Chemistry* 2015, *17* (4), 2418–2425. https://doi.org/10.1039/c4gc02519a.

(9) Wu, J.; Wang, X.; Song, J.; Wang, Q.; Shi, L.; Wang, X.; Huo, M. Synthesis of Heteropolyacid (HPA) Functionalized Graphitic Carbon Nitride as Effective Catalysts for Converting Polysaccharides into High-Value Chemicals. *Resour Conserv Recycl* 2022, *185*. https://doi.org/10.1016/j.resconrec.2022.106473.
